# Rise and fall of subclones from diagnosis to relapse in pediatric B-acute lymphoblastic leukaemia

**DOI:** 10.1038/ncomms7604

**Published:** 2015-03-19

**Authors:** Xiaotu Ma, Michael Edmonson, Donald Yergeau, Donna M. Muzny, Oliver A. Hampton, Michael Rusch, Guangchun Song, John Easton, Richard C. Harvey, David A. Wheeler, Jing Ma, HarshaVardhan Doddapaneni, Bhavin Vadodaria, Gang Wu, Panduka Nagahawatte, William L. Carroll, I-Ming Chen, Julie M. Gastier-Foster, Mary V. Relling, Malcolm A. Smith, Meenakshi Devidas, Jaime M. Guidry Auvil, James R. Downing, Mignon L. Loh, Cheryl L. Willman, Daniela S. Gerhard, Charles G. Mullighan, Stephen P. Hunger, Jinghui Zhang

**Affiliations:** 1 Computational Biology, St Jude Children’s Research Hospital, Memphis, Tennessee 38105, USA; 2 Pediatric Cancer Genome Project Validation Lab, St Jude Children’s Research Hospital, Memphis, Tennessee 38105, USA; 3 Cancer Genomics, Human Genome Sequencing Center, Baylor College of Medicine, Houston, Texas 77030, USA; 4 Pathology, St Jude Children’s Research Hospital, Memphis, Tennessee 38105, USA; 5 University of New Mexico Cancer Center, Albuquerque, New Mexico 87131, USA; 6 Perlmutter Cancer Center, NYU Langone Medical Center, New York, NY 10016, USA; 7 Department of Pathology and Laboratory Medicine, Nationwide Children's Hospital, Columbus, Ohio 43205, USA; 8 Departments of Pathology and Pediatrics, The Ohio State University, Columbus, Ohio 43210, USA; 9 Department of Pharmaceutical Sciences, St Jude Children’s Research Hospital, Memphis, Tennessee 38105, USA; 10 Cancer Therapy Evaluation Program, National Cancer Institute, Bethesda, Maryland 20892, USA; 11 Department of Biostatistics, Colleges of Medicine, Public Health & Health Professions, University of Florida, Gainesville, Florida 32607, USA; 12 Office of Cancer Genomics, National Cancer Institute, Bethesda, Maryland 20892, USA; 13 Department of Pediatrics, Benioff Children’s Hospital and the Helen Diller Family Comprehensive Cancer Center, University of California San Francisco, San Francisco, California 94143, USA; 14 Division of Oncology and The Center for Childhood Cancer Research, Children’s Hospital of Philadelphia, Philadelphia, Pennsylvania 19104, USA

## Abstract

There is incomplete understanding of genetic heterogeneity and clonal evolution during cancer progression. Here we use deep whole-exome sequencing to describe the clonal architecture and evolution of 20 pediatric B-acute lymphoblastic leukaemias from diagnosis to relapse. We show that clonal diversity is comparable at diagnosis and relapse and clonal survival from diagnosis to relapse is not associated with mutation burden. Six pathways were frequently mutated, with *NT5C2*, *CREBBP*, *WHSC1*, *TP53*, *USH2A*, *NRAS* and *IKZF1* mutations enriched at relapse. Half of the leukaemias had multiple subclonal mutations in a pathway or gene at diagnosis, but mostly with only one, usually minor clone, surviving therapy to acquire additional mutations and become the relapse founder clone. Relapse-specific mutations in *NT5C2* were found in nine cases, with mutations in four cases being in descendants of the relapse founder clone. These results provide important insights into the genetic basis of treatment failure in ALL and have implications for the early detection of mutations driving relapse.

## Abstract (web summary)

Genetic heterogeneity and clonal evolution contribute to cancer progression. Here Ma *et al.* use deep whole-exome sequencing to identify recurrently mutated pathways and clonal architecture in pediatric acute lymphoblastic leukaemia, shedding light on the evolutionary trajectory from diagnosis to relapse

## Graphical abstract

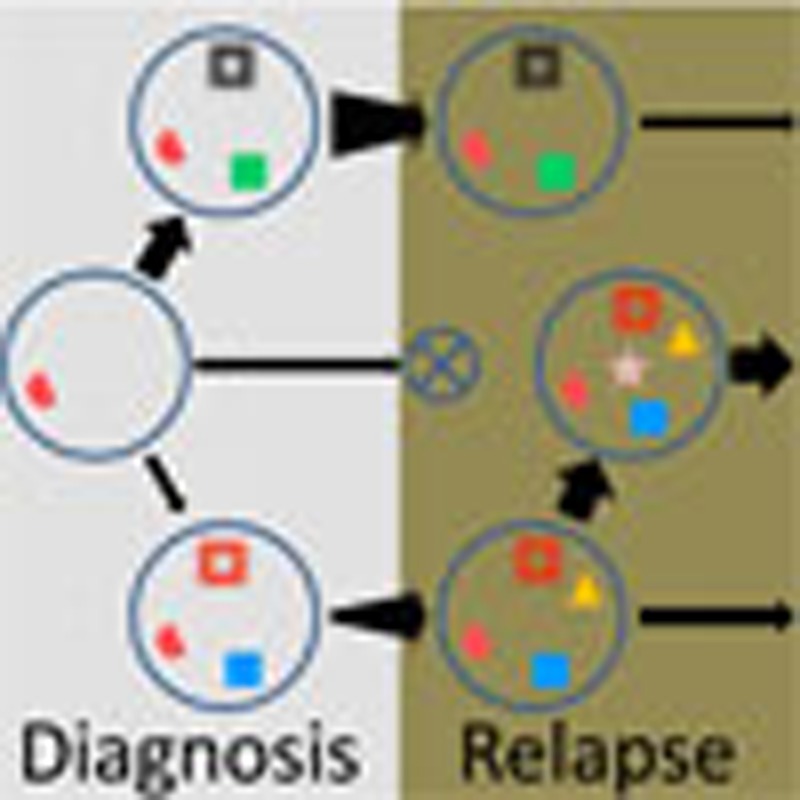

Despite event-free survival rates for pediatric acute lymphoblastic leukaemia (ALL) that now exceed 85%, about 15% of children with ALL experience disease recurrence, most of whom will die, and relapsed ALL remains a leading cause of cancer-related death in children^1^,^2^,^3^,^4^. Recent genomic studies have identified relapse-specific mutations in pediatric ALL^5^,^6^,^7^ and have constructed clonal lineage from diagnosis to relapse for several cancers^8^,^9^,^10^. These studies have provided insight into tumour heterogeneity and the evolutionary trajectory from diagnosis to relapse. Most studies have focused primarily on genetic lesions present in the clones that survive therapy (that is, the rising clones) but not those eradicated by therapy (that is, the falling clones)^8^,^10^. While the technology does not exist to prove complete eradication of a clone, in this study we use the term ‘eradication’ to refer to clones that are no longer detectable within the limits of the assays. In addition to discovering genetic lesions responsible for relapse, deep genome-wide sequencing of matched samples obtained at diagnosis, remission and relapse has the potential to characterize evolutionary lineages and to address key issues in tumour clonal evolution. These include the uniqueness of genetic lesions in rising clones that persist to relapse; the relative mutation burden of rising and falling clones; whether or not the rising clone at relapse always arises from a minor clone at diagnosis; and the chronology of clonal emergence and mutation acquisition at diagnosis and relapse.

To investigate how genetic lesions contribute to the rise and fall of subclones from diagnosis to relapse in childhood B-ALL, we analyse somatic sequence mutations, structural variations (SVs) and DNA copy-number alterations of samples obtained at diagnosis, remission and relapse from 20 children with B-progenitor ALL (B-ALL) studied as a part of a collaborative study from the Children’s Oncology Group (COG), the National Cancer Institute Therapeutically Applicable Research to Generate Effective Treatments (TARGET) initiative and the St Jude–Washington University Pediatric Cancer Genomic Project. The median age of the patients at diagnosis was 7 years (range 2 to 19). Cases were selected for analysis based on the occurrence of an early bone marrow relapse (<36 months; median 19.2 months, range 3.8 to 35.7), which is associated with very poor survival^11^. High-coverage whole-exome sequencing (WXS) followed by deep sequencing of somatic variants identified at each time point in the trio samples unveils the characteristics of rising and falling subclones from diagnosis to relapse in this group of high-risk pediatric B-ALL.

## Results

### Somatic mutation profile at diagnosis and relapse

We performed WXS at high coverage (~200-fold) of samples obtained at diagnosis, remission and relapse from 20 patients treated on recent COG B-ALL trials (Methods, Supplementary Data 1 and Supplementary Fig. 1). Eleven cases were found to harbour known oncogenic gene fusions and rearrangements including *TCF3-PBX1* (*n*=5), *TCF3-HLF* (*n*=1), *IGH-CRLF2* (*n*=3), *P2RY8-CRLF2* (*n*=1) and *ETV6-RUNX1* (*n*=1) at diagnosis, all of which were retained at relapse. In addition to sequence mutations (that is, single-nucleotide variation (SNV) and insertion/deletion (indel), somatic SV and copy-number variation (CNV) were identified by analysis of whole-genome sequencing (WGS) and single-nucleotide polymorphism (SNP) array data. A total of 2,278 SNVs, 106 indels and 68 SVs were experimentally verified (Supplementary Data 2 and 3) by capture sequencing of diagnosis, relapse and remission DNA at an average of 696-fold coverage (Methods). The mutant allele fraction (MAF) values obtained from WXS showed high concordance with those from capture sequencing (Pearson’s correlation coefficient was 0.973 and the mean difference in MAF between WXS and capture sequencing was 0.007 (Supplementary Fig. 2)). The combined coverage of WXS and capture was 883-fold, which had the power to detect over 99% of the mutations with a MAF of at least 0.01. This represented the lower limit used for determining the presence of a mutant allele throughout this study (Methods). Variants below this level are referred as relapse- or diagnosis specific, as appropriate, with the caveat that failure to detect some could potentially be caused by limitations of technology and/or read depth. Seventy-two per cent of the mutations had MAF of <0.3, and were thus likely subclonal.

Each case had genetic alterations shared at diagnosis and relapse, including the chimeric gene fusions described above, indicating a common origin from a preleukaemic ancestral clone (Supplementary Fig. 3), consistent with previous SNP array data in B-ALL^9^. On average, 74% (range 14–100%) of the coding sequence mutations present at diagnosis persisted to relapse. Four relapsed tumours were hypermutable with 156–740 coding mutations, two of which had loss-of-function mutations in the DNA mismatch repair genes *PMS2* and *MSH6* at relapse. Three of these hypermutable cases had substantial changes in their mutational spectra, with the prevalence of transition mutations increasing from 60–70% at diagnosis to over 95% at relapse (Supplementary Fig. 4). For the remaining 16 non-hypermutable cases, the number of coding somatic sequence mutations at relapse (median 31, range 5–59) was significantly higher (*P*=0.004, Wilcoxon signed-rank test) than that at diagnosis (median 18, range 6–48). No case had a significant change in the number of SVs and CNVs at relapse. Across the entire cohort, 78% of CNVs (Supplementary Fig. 3b) and 82% of SVs present at diagnosis persisted to relapse, while 40% of CNVs and 7% of SVs were relapse specific (Supplementary Data 3 and 4). Although chemotherapy may have directly induced mutations observed at relapse, the limited exposure to known mutagenic agents in the chemotherapeutic regimens used, and the lack of substantial increases in sequence mutation burden or shift in mutation spectrum suggests this was not an important mechanism in the acquisition of mutations in the majority of cases.

### Recurrently mutated genes/pathways at diagnosis and relapse

**Figure 1:**
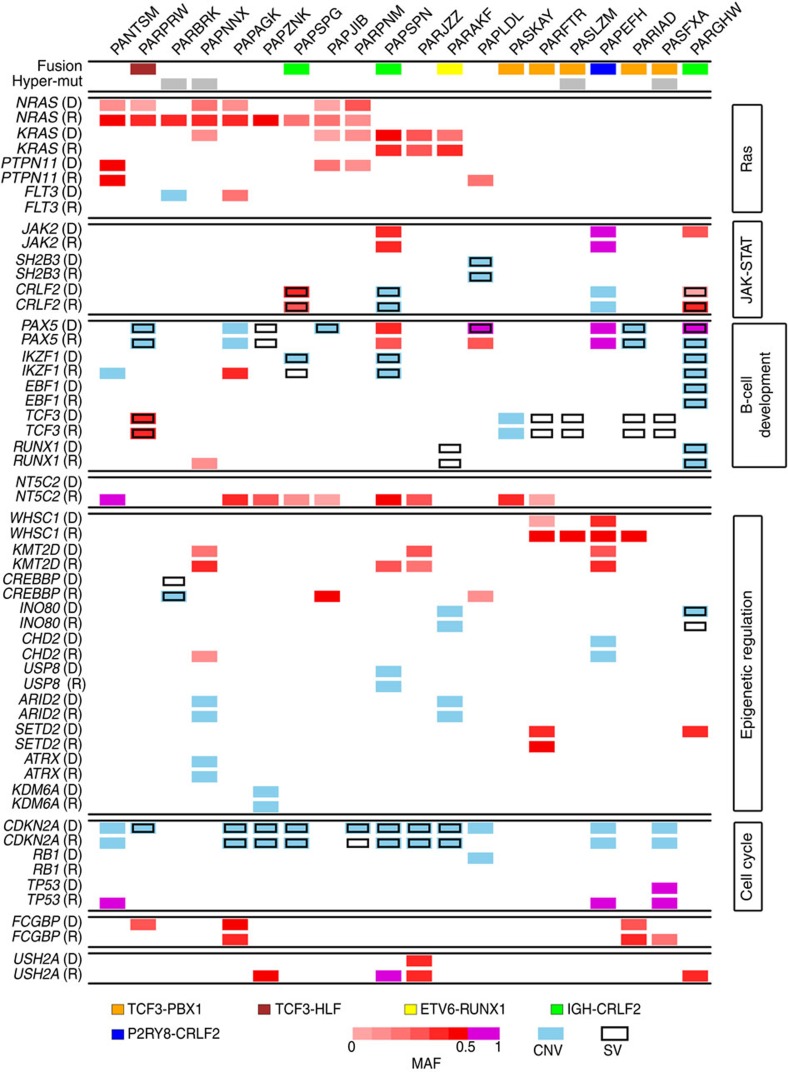
Recurrently mutated pathways and genes in 20 ALL trios. Mutations present in diagnosis (D) and relapse (R) tumour of each case are shown in two rows. SNVs and indels are shown in red or magenta in shades that match their MAF indicated by the colour scale. Copy-number alterations (including deletion and amplification) are shown in cyan, while SVs are represented by black open boxes. Presence of both SVs and CNVs in the same gene depicts a single lesion in which SV breakpoints determined by sequencing data corroborates CNV segment boundary. Cases with oncogenic fusions involving *TCF3*, *ETV6*, *RUNX1* and *CRLF2* are marked at the top along with hypermutators having over 100 SNVs/indels at relapase. SV data for PANTSM, PAPNNX, PASKAY and PAPEFH are not available as they were not analysed by WGS.

Integrated analysis of sequence mutations, SVs and CNVs identified six pathways mutated at high frequency at diagnosis and/or relapse: Ras signalling (65%), JAK-STAT signalling (25%), transcriptional regulation of lymphoid development (85%), nucleoside metabolism (45%), epigenetic modification (65%) and cell cycle regulation (60%; Fig. 1). The frequency of Ras pathway mutations was similar at diagnosis (50%) and relapse (65%). However, a notable finding was the presence of multiple subclonal Ras mutations, either in the same gene (*NRAS*, *KRAS*) or in different Ras pathway genes (*KRAS*, *NRAS*, *PTPN11*, *FLT3*) in nine cases at diagnosis (Fig. 1 and Supplementary Data 2). In five out of the nine cases, we observed convergence from multiple subclonal mutations in *NRAS*, *KRAS*, *PTPN11* and *FLT3* at diagnosis to a single clonal *NRAS* mutation at relapse (PARPRW, PAPNNX, PAPAGK, PAPJIB, PARPNM; Fig. 1 and Supplementary Data 2). For example, PAPJIB had subclonal mutations of KRAS p.Ala146Thr, NRAS p.Gly13Asp and PTPN11 p.Ser502Pro with MAFs of 0.021, 0.025 and 0.233, respectively, at diagnosis, and persistence of only the NRAS p.Gly13Asp mutation with MAF increased 10-fold to 0.234 at relapse. SNVs and SVs resulting in activation of the JAK signalling pathway were present in 25% of the cases at diagnosis and relapse. Loss-of-function mutations, mostly SVs and CNVs, in genes encoding regulators of B-cell development, were present in 75 and 80% of the cases at diagnosis and relapse, respectively. Cell cycle pathway alterations were present in 60% of the tumours at diagnosis and 50% of the tumours at relapse. This included two cases with diagnosis-specific *CDKN2A/B* deletions (10 cases had deletions at both diagnosis and relapse) and two cases with relapse-specific *TP53* sequence mutations.

Sequence mutations in *NT5C2*, a gene encoding a 5′-nucleotidase involved in purine metabolism, were observed exclusively at relapse in 45% of the cases, including known activating mutations in relapsed ALL^6^,^7^ and a novel recurrent mutation p.Arg39Gln (Supplementary Fig. 5a). The mutation rate of epigenetic regulators was 45 and 65% at diagnosis and relapse, respectively, with recurrent relapse-specific mutations found in *WHSC1* (*n*=2) and *CREBBP* (*n*=2; Supplementary Fig. 5b,d). Two lesions in this pathway were detected at very low level at diagnosis but highly enriched in relapse. The WHSC1 p.Glu1099Lys mutation in case PARFTR exhibited an increase in MAF from 0.01 at diagnosis to 0.49 at relapse, while an intragenic deletion of *CREBBP* in PARBRK, detectable at diagnosis only by high-coverage capture sequencing, was found to be clonal at relapse by analysis of WGS and SNP array data. In addition to these six pathways, frequent non-silent sequence mutations were found in *FCGBP* (immunoglobulin Fc gamma-binding protein; *n*=4) and *USH2A* (mutated in Usher syndrome and retinitis pigmentosa; *n*=4; Supplementary Fig. 5c).

**Figure 2:**
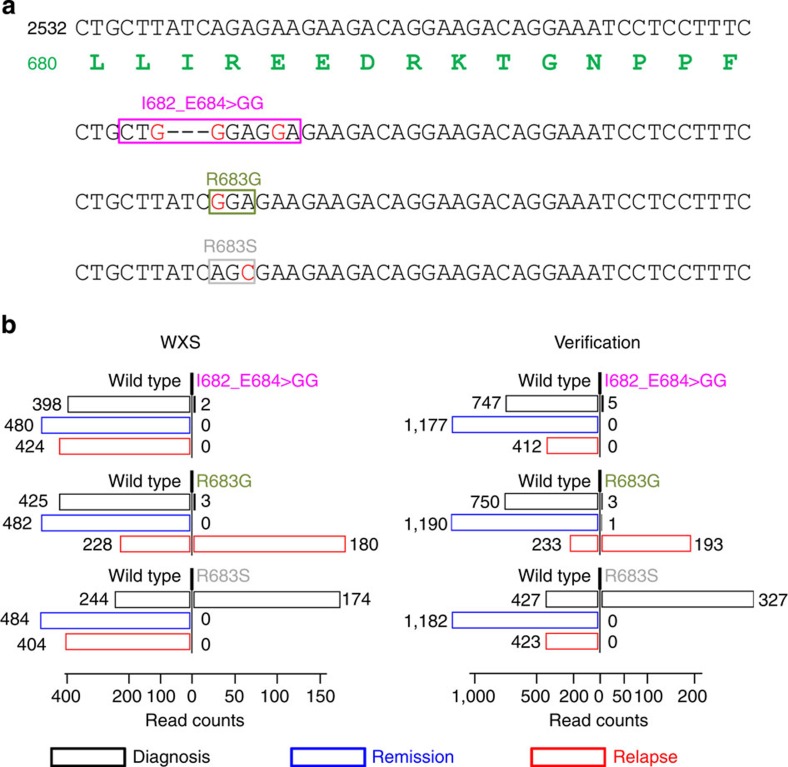
Three subclonal *JAK2* mutations in patient PAPSPN. (**a**) Alignment of sequencing reads that harbour the three mutations present at diagnosis. (**b**) Sequencing read count of mutant (to the right of *y* axis) and wild-type (to the left of *y* axis) alleles of the three mutations categorized into diagnosis (black), remission (blue) and relapse (red) classes in the discovery sequencing (WXS, left) and verification sequencing (right). Colour code of mutations matches that of **a**.

Multiclonal mutations were observed in six genes in 10 cases at diagnosis or relapse: *NT5C2* (*n*=3), *KRAS* (*n*=3), *NRAS* (*n*=2), *JAK2* (*n*=2), *CREBBP* (*n*=1) and *PAX5* (*n*=1) (Supplementary Data 5). Multiclonal mutations in *NT5C2* and *CREBBP* were relapse specific, while multiclonal mutations in *KRAS*, *NRAS*, *JAK2* and *PAX5* at diagnosis exhibited variable persistence. Case PAPSPN is illustrative of the dynamic population changes of multiclonal mutations. This case had multiclonal mutations in three genes (*JAK2*, *KRAS* and *PAX5*), all of which showed switching of the predominant mutation site from diagnosis to relapse. Three mutations affecting JAK2 p.Arg683, a known hotspot of mutation in B-ALL^12^,^13^,^14^, were present at diagnosis (Fig. 2a). JAK2 p.Arg683Ser was the predominant clonal mutation (MAF=0.42), while p.Arg683Gly (MAF=0.007) and p.Ile682_Glu684>GlyGly (MAF=0.005) were minor subclonal mutations (Fig. 2a). Only JAK2 p.Arg683Gly persisted to relapse to become a clonal mutation with MAF of 0.44. Importantly, the MAFs detected by WXS were verified by deep sequencing (Fig. 2b). Similarly, the dominant clonal mutation in KRAS switched from p.Gly12Asp at diagnosis to p.Ala146Thr at relapse, while the frameshift mutation PAX5 p.Glu97fs at diagnosis was replaced by a relapse-specific in-frame insertion p.Gly48>ValMetIleIleLysValSer. In addition to sequence mutations, we also detected multiclonal SVs in *PAX5* (*n*=2) and *CDKN2A* (*n*=1; Supplementary Figs 6,7 and 16).

### Clonal evolution from diagnosis to relapse

**Figure 3:**
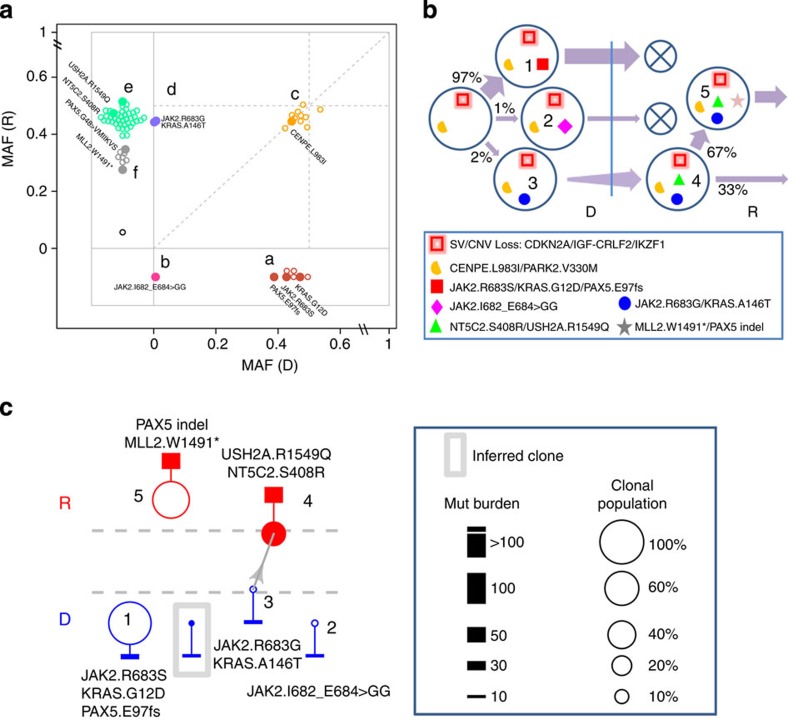
Clonal architecture of diagnosis and relapse samples for PAPSPN. (**a**) Six mutation clusters (marked *a*–*f*) constructed from MAF at diagnosis (D; *x* axis value) and relapse (R; *y* axis value). Shared mutations are shown in the large square in the centre, while the rectangular areas at the bottom and the left display D- and R-specific mutation clusters, respectively. Each mutation cluster is highlighted in colour with individual mutation marked by an open dot. A mutation in a key pathway is labelled and marked by a solid dot. The MAFs are not normalized by tumour purity. (**b**) Clonal lineages at diagnosis and relapse. Each clone is identified with a number. Mutation clusters present in each clone are marked by distinct shapes in colours that match those in **a**. Key mutations as well as CNVs and SVs in each cluster are labelled in the legend. D and R are demarcated by a vertical blue bar. Clonal population size is labelled as percentage of tumour content. The thickness of an arrow from a progenitor clone to its descendant is proportional to population change. ‘Falling’ clones that did not survive therapy are marked by an X. (**c**) Pattern of rise and fall of subclones from diagnosis to relapse. The legend for the symbols is shown at the right. Remission was marked by dashed lines that separate diagnosis and relapse. Each clone is shown by a circle drawn in proportion to its population size and attached with a bar whose thickness is proportional to mutation burden. A founder clone at D or R is indicated by a solid circle. An inferred founder with population frequency below the detection level (<1%) is marked by a grey box. The clones at D and R are shown in blue and red, respectively. Representive mutations specific to each clonal lineage are marked. A rising clone in relapse that survived therapy is connected to its progenitor at diagnosis by a line.

**Figure 4:**
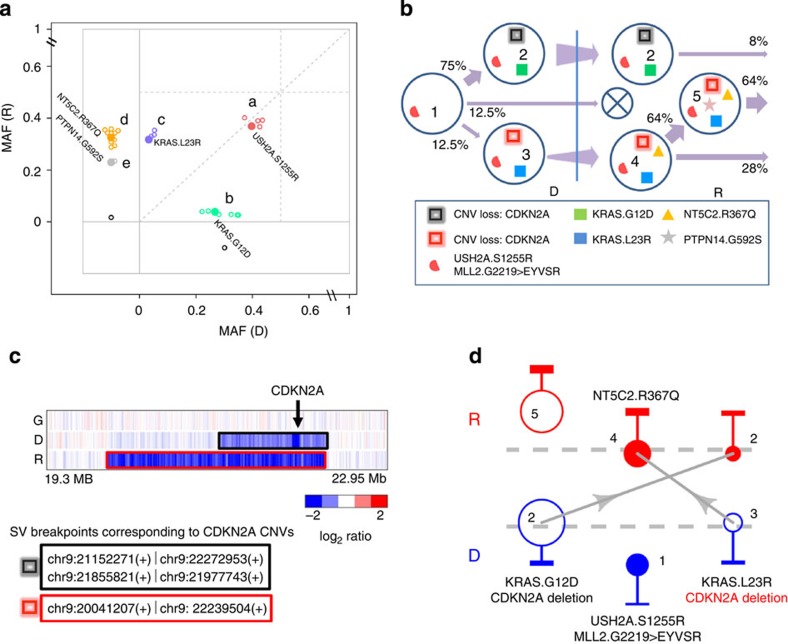
Clonal architecture of diagnosis and relapse samples for PARJZZ. (**a**) Five mutation clusters (marked *a*–*e*) constructed from MAF at diagnosis (D) and relapse (R). (**b**) Clonal lineages from diagnosis to relapse. Clone 2 harbours cluster *b* mutation labelled as KRAS p.Gly12Asp (green solid box) and *CDKN2A* focal deletion (black open box). Clone 3 harbours cluster *c* mutations labelled as KRAS p.Lys23Arg (blue solid box) and a different *CDKN2A* focal deletion (red open box). (**c**) Probe intensity of SNP array in germline (G), diagnosis (D) and relapse (R) for the genomic locus (chr9:19.3Mb-22.95Mb) of *CDKN2A*. Intensity of red and blue colour corresponds to the log_2_ ratio shown in the legend. The predominant *CDKN2A* lesions at diagnosis were two deletions marked by a black rectangle, while the predominant deletion at relapse is marked by a red rectangle and detectable at diagnosis in a subclone as indicated by the light-blue shaded regions in the diagnostic tumour. The genomic locations of the SVs encompassing the three deletions are shown below. (**d**) Dual lineage of the relapsed tumour with both clone 2 and clone 3 from diagnosis persisted to relapse but switched their clonal dominance.

**Figure 5:**
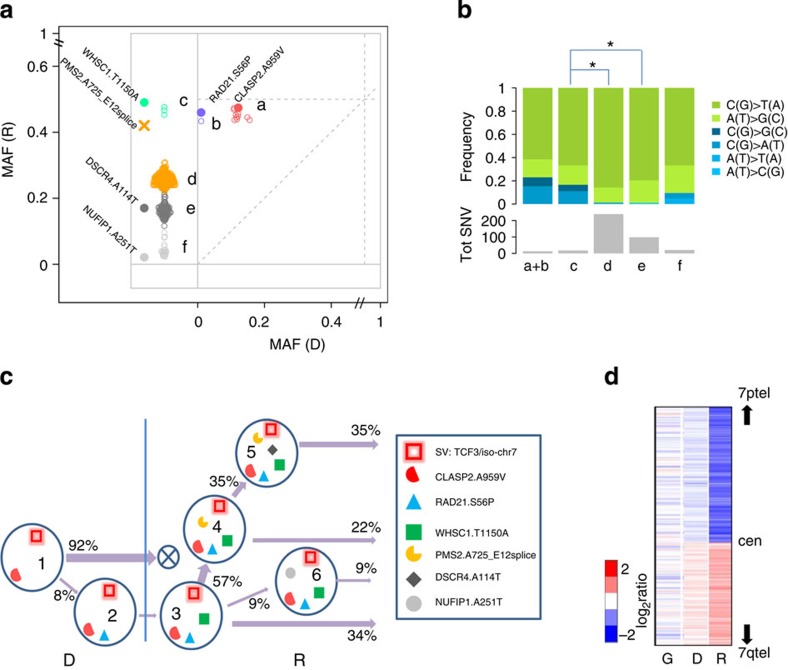
Clonal architecture of diagnosis and relapse samples for case PASLZM. (**a**) Six mutation clusters (marked *a*–*f*) constructed from MAF at diagnosis (D) and relapse (R). The splice mutation in PMS2 (A725_E12splice) is placed to cluster *d* because it is located on a haploid region due to loss of chromosome 7p at relapse. (**b**) Comparison of mutation spectra of relapse-specific clusters (*c*–*f*) and all diagnosis (*a*+*b*) mutations. Relapse-specific clusters (*d*,*e*) show significantly higher (*P*<0.05, Fisher’s exact test; indicated by an asterisk) transition mutation rates (and mutation counts) than clusters *c* and *a*+*b*. (**c**) Clonal lineages from diagnosis to relapse and the ambiguous lineage of cluster *e*,*f* are resolved with the input from mutation spectrum analysis. Specifically, clone 5, which contains mutation cluster *e* with a total of 98 SNVs at a MAF of 0.15, can be considered as a descendant of either clone 4 or clone 3. As the mutation spectrum for cluster *e* is highly enriched for transition changes, consistent with cluster *d* mutations in clone 4 but significantly different (*P*=0.01, Fisher’s exact test) from that of cluster *c* mutations in clone 3, it is placed as a descendant of clone 4 instead of clone 3. Clone 6 contains 21 lineage-specific mutations of cluster *f* with a MAF of ~0.05. It would have an ambiguous clonal linage that could have descended from clone 3, or clone 4 or clone 5 if compatibility of population frequency is the only criteria for determining lineage. It was placed under clone 3 as its mutation spectrum is significantly different from that of clones 4 and 5 but not from that of clone 3. (**d**) Probe intensity of SNP array in germline (G), diagnosis (D) and relapse (R) on chromosome 7. Isochromosome 7q (that is, loss of 7p and gain of 7q) demonstrated a low signal intensity due to low tumour purity at diagnosis and is present in all tumour cells at relapse.

In light of the observed turnover of predominant driver mutations in genes or pathways (Figs 1 and 2), we used deep sequencing data derived from high-coverage WXS and mutation verification data to define clonality. We constructed clonal lineages based on frequency of mutation clusters at diagnosis and relapse^8^. To improve the precision of the analysis, we developed a binomial mixture model that explicitly accounts for read coverage and determines mutation clusters in three categories: diagnosis specific, relapse specific and shared (see Methods). Clonal architecture for each case is presented in Figs 3, 4, 5 and Supplementary Figs 7–23. Dynamic changes of subclonal population sizes were observed in most of the cases. Here we present three diverse examples to illustrate how clonal evolution resulted in the turnover of the predominant mutations in signalling pathways, the reversal of clonal dominance in a dual-lineage evolution and changes of mutation spectrum after acquiring a relapse-specific mutation in a DNA mismatch repair gene. In these analyses, we refer to three complementary concepts that incorporate MAF, overall mutation load and biological processes at each time point: clusters that use relative MAF at diagnosis and relapse to aggregate multiple mutations; clones that contain multiple clusters constructed by mathematical modelling and lineages that trace clonal rise and fall from ancestral, prediagnosis clones through diagnosis and relapse.

Case PAPSPN illustrates how clonal evolution resulted in the turnover of the predominant mutations in genes involved in multiple cellular pathways, including JAK-STAT signalling (*JAK2*), Ras signalling (*KRAS*) and lymphoid development (*PAX5*) as well as acquisition of relapse-associated mutations in epigenetic regulators (*MLL2*) and drug-metabolizing genes (*NT5C2*). Combining data at diagnosis and relapse, a total of six mutation clusters were identified (*a*–*f*, Fig. 3a). Clusters *a* and *b* were diagnosis specific; clusters *c* and *d* were shared in diagnosis and relapse; and clusters *e* and *f* were relapse specific. At diagnosis, the mutation clusters *a* and *c* have high MAF suggesting that they are present in nearly all tumour cells at diagnosis, while the mutation clusters *b* and *d* had low MAFs (~0.01) suggesting that they were present in a small fraction of tumour cells. Clonal identity was established from MAF and tumour specificity of mutation cluster as MAF of a cluster is expected to match the combined population frequency of all clones that contain this mutation cluster (Methods). For PAPSPN, three clones (designated Clone 1–3 in Fig. 3b) were identified at diagnosis; all evolved from a founder clone that had cluster *c* mutations along with focal deletions in *CDKN2A/B*, *IKZF1* and *IGH-CRLF2* fusion. Mutations in a founder clone were expected to be present in all tumour cells at diagnosis and relapse, which matched the MAF profile of cluster *c* mutations. Cluster *a* had comparable MAF as cluster *c* but was diagnosis specific, suggesting the presence of a predominant clone (clone 1) that had both cluster *a* and cluster *c* mutations. The founder clone that was comprised exclusively of cluster *c* mutation was inferred as the current sequencing depth would not permit differentiating the MAF of cluster *a* from that of cluster *c* at diagnosis. The cluster *a* mutations defined the identity for the predominant clone 1 and they included JAK2 p.Arg683Ser, KRAS p.Gly12Asp and PAX5 p.Glu97fs (MAF 0.455). Clones 2 and 3 were minor clones, accounting for ~1 and 2% of the tumour cells at diagnosis, respectively. The lineage-specific mutations in clone 2 and clone 3 were contained in cluster *b* (which included the JAK2 p.IleI682_Glu684>GlyGly) and cluster *d* (which included JAK2 p.Arg683Gly), respectively. All JAK2 mutations at this residue result in constitutive JAK-STAT activation and are likely key driver mutations in ALL^13^. Under the assumption that activating mutations affecting the same pathway are mutually exclusive, we assigned each JAK2 mutation to a distinct subclone. Of these three clones present at diagnosis, only clone 3 survived chemotherapy to become the progenitor for the two subclones (designated clone 4 and clone 5 in Fig. 3b) detected at relapse. Clone 4 was the direct descendant of clone 3 as its lineage-specific mutations in cluster *e* (including NT5C2 p.Ser408Arg and USH2A p.Arg1549Gln along with 36 other SNVs) had the same MAF as the cluster *c* and *d* mutations ‘inherited’ from clone 3. Analysis of coverage and MAF showed that cluster *e* was likely distinct from cluster *d* despite the low MAF of cluster *d* at diagnosis (*P*=2 × 10^−9^, binomial test; Methods). Clone 5 evolved from clone 4 by acquiring additional subclonal mutations in cluster *f* which include MLL2 p.Trp1491* and PAX5 p.Gly48>ValMetIleIleLysValSer. The projected population sizes of clones 4 and 5 at relapse were 33 and 67%, respectively. As a result, the loss of clone 1 coupled with the expansion of clone 3 resulted in switching of the predominant JAK2 mutation from p.Arg683Ser in diagnosis to p.Arg683Gly in relapse.

PARJZZ is an illustrative case that has two rising clones from diagnosis to relapse, and was the only case exhibiting this pattern in this cohort. Of the five mutation clusters (*a*–*e*, Fig. 4a) identified in this case, the ancestral clone (clone 1) had mutations of cluster *a* and constituted 12.5% of the tumour cells at diagnosis. Clone 2 and clone 3, both of which were descendants of clone 1, contained mutations from cluster *a* along with their respective lineage-specific mutations in cluster* b* (represented by KRAS p.Gly12Asp mutation) and *c* (represented by KRAS p.Leu23Arg). In addition to having distinct sequence mutations in KRAS, these two clones also harboured distinct homozygous deletions of *CDKN2A/B* (Fig. 4b,c, Supplementary Fig. 6). Clone 2 had homozygous deletion of *CDKN2A/B* arising from two independent deletions of 1.1 and 0.1 Mb in size. Clone 3 had a 2.2-Mb homozygous deletion of *CDKN2A/B* caused by a deletion followed by copy-neutral loss-of-heterozygosity at chromosome 9 (hg19 coordinates 20,041,207–22,239,504; Supplementary Fig. 6). Both clone 2 and clone 3 persisted to relapse but with reversal of clonal dominance (Fig. 4d). Clone 2 was the predominant clone at diagnosis and accounted for 75% of the tumour cells, but represented a minor clone at relapse with only 8% of the tumour cells. On the other hand, the two descendants of clone 3, a minor subclone at diagnosis that accounted for 12.5% of the tumour cells, had a combined population frequency of 92% at relapse after acquiring a relapse-specific NT5C2 mutation p.Arg367Gln.

Case PASLZM was one of the four cases with hypermutation at relapse. A total of six mutation clusters were identified (Fig. 5a), four (*c*–*f*) of which were relapse specific. Clone 1 was the predominant ancestral clone, while clone 2 was its minor descendant that accounted for 8% of leukaemia cells at diagnosis (Fig. 5c). In addition to *TCF3-PBX1* fusion, clone 1 harbours a clonal isochromosome 7q detectable only by manual analysis of SNP array probe intensity data due to low tumour purity of the diagnostic sample (Fig. 5d and Supplementary Data 1). The lineage-specific mutation cluster for clone 2 was cluster *b*, which included a RAD21 p.Ser56Pro mutation. Clone 2 persisted to relapse and its descendant, clone 3, acquired a WHSC1 SET domain mutation p.Thr1150Ala along with four other mutations in cluster *c*. Despite the low MAF of cluster *b* at diagnosis, statistical analyses established that cluster *c* was clearly distinct from cluster *b* (*P*=3 × 10^−7^, binomial test; Methods). Clone 4 descended from clone 3 after acquiring an additional 239 mutations in cluster *d*. This cluster included a somatic splice site mutation of *PMS2*, which encodes post-meiotic segregation increased 2, a protein involved in mismatch repair that is mutated in hereditary non-polyposis colorectal cancer (Lynch syndrome) and other tumours^15^. Notably, while the MAF of the *PMS2* mutation was 0.43, we assigned this mutation to cluster *d* (whose MAF centres on 0.26) rather than cluster *c* because *PMS2* is located at 7p, which is hemizygous at relapse due to clonal isochromosome 7q. Therefore, the *PMS2* mutation is subclonal as the high MAF was caused by loss-of-heterozygosity at 7p. Interestingly, it is an exact match to the germline mutation c.2174+1G>A ( http://www.ncbi.nlm.nih.gov/clinvar/RCV000076844) known to be pathogenic in Lynch syndrome^15^. Transition events account for 99% of the SNVs in cluster *d* compared with 77 and 83% of SNVs observed at diagnosis and cluster *c*, consistent with the biallelic loss of *PMS2* in clone 4. Ambiguity in the lineage of clone 5 and clone 6 was resolved by similarity of mutation spectrum (Fig. 5b). Together these observations suggest that the *PMS2* mutation is directly responsible for the hypermutator phenotype of this relapse case, and represents a new mechanism of mutation acquisition in relapsed ALL.

### Rise and fall of subclones from diagnosis to relapse

**Figure 6:**
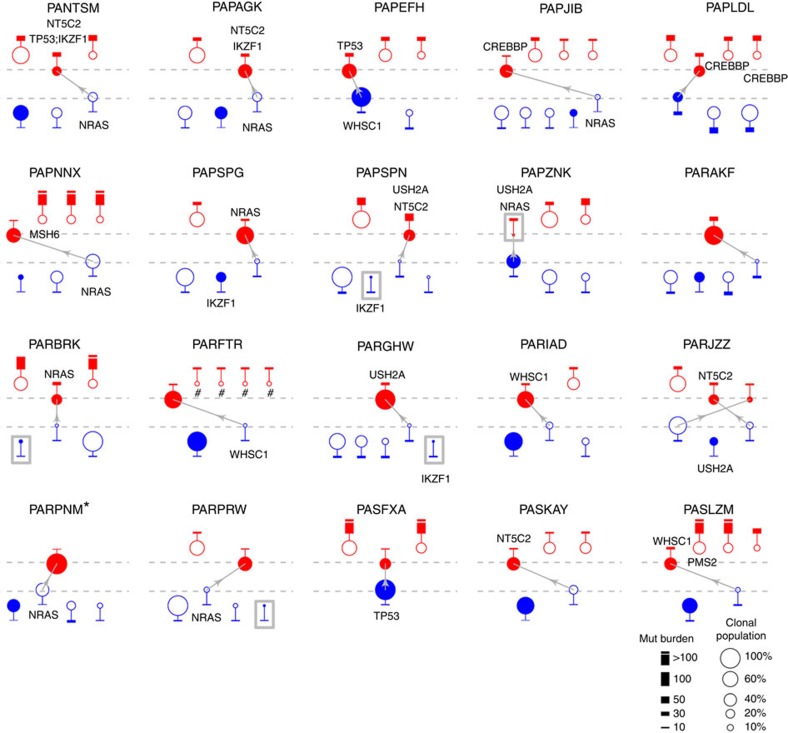
The pattern of rise and fall of subclones from diagnosis to relapse of the 20 cases analysed in this study. PARPNM is marked by an asterisk (*) due to potential underestimation of clonal diversity at relapse due to low tumour purity (<20%). The graphic representation is the same as Fig. 3c. The four relapse subclones in PARFTR harbour distinct NT5C2 mutations and are marked with a #. Subclones which acquired (but not inherited) mutations in the seven relapse-enriched genes and DNA mismatch repair genes are labelled with the corresponding gene symbols. PAPEFH, PAPNNX, PAPZNK, PARPNM and PASFXA are the five cases in which the rising clone originated from a predominant clone at diagnosis.

Using clonal lineage data for the 20 cases, we evaluated how clonal diversity, origin, mutation burden and population frequency contributed to clonal evolution from diagnosis to relapse (Fig. 6). There was no significant difference (*P*=0.6; Wilcoxon signed-rank test) between the median number of subclones identified at diagnosis (3; range 1–5) and at relapse (3; range 1–5). In general, the numbers of mutations in the rising clones (median 11, range 2–32) were comparable (*P*=0.7, Wilcoxon rank-sum test) to the numbers present in the falling clones (median 11, range 1–44) indicating that clonal survival is not dependent on mutation burden. In 15 of 20 cases (75%), the rising clone was a minor subclone at diagnosis (median population frequency 7%, range 2–20%). Relapse-specific mutations present in the founder clone at relapse were observed in the following seven genes: *NT5C2* (*n*=5), *USH2A* (*n*=3), *WHSC1* (*n*=2), *TP53* (*n*=2), *NRAS* (*n*=3), *IKZF1* (*n*=2) and *CREBBP* (*n*=2). They were observed to be relapse specific (*NT5C2*), enriched at relapse (*CREBBP*, *USH2A*, *WHSC1*, *IKZF1*, *NRAS* and *TP53*) or to have increased mutation burden at relapse (*NRAS*, *WHSC1* and *CREBBP).* Mutations in these genes were never present in the lineage of falling clones except for *NRAS* mutations that exist in multiple subclones at diagnosis. Nineteen cases had mutations in at least one of these genes and nine cases had mutations in multiple of these genes. Except for *CREBBP* and *WHSC1*, both of which affect epigenetic regulation, the relapse-enriched genes were not mutually exclusive at the case or clonal level as they affect different biological processes. Of the five cases that had predominant clones at diagnosis that persisted to relapse (PAPEFH, PAPNNX, PAPZNK, PARPNM and PASFXA), three had rising clones that constituted ~50% of the tumour cells at diagnosis and the other two (PAPEFH and PASFXA) had major clones being the rising clones. Interestingly, these two cases harboured mutations in genes that would otherwise be considered relapse-specific: PASFXA has a homozygous TP53 p.Arg248Gln mutation and PAPEFH has WHSC1 p.Glu1099Lys in the predominant clone at diagnosis.

## Discussion

We employed deep sequencing by WXS and by custom capture of 20 diagnosis–relapse–remission samples of pediatric B-ALL at an average coverage of 200- and 700-fold, respectively, to gain insight into the genetic and clonal architecture of relapsed ALL. At this sequencing depth, our analysis has the power for detecting 92% of the sequence mutations at MAF≥0.03 by WXS. Combining WXS and capture data enabled the identification of 99% of mutations at MAF≥0.01 if the mutations were detected in one tumour but missed in the other for the same case (Methods). Among the 116 subclones identified in this cohort, six from relapsed leukaemias (cases PANTSM, PARBRK, PAPNNX, PAPEFH, PASFXA, PASKAY) and one diagnosis ALL case (PAPZNK) had ambiguous clonal lineages. In each case, a diagnosis- or relapse-specific mutation cluster of the smallest frequency resulted in ambiguity in clonal lineage assignment and none involved genes in the six frequently mutated pathways (Fig. 1). As a result, this ambiguity does not affect clonal population size or mutation turnover analyses that describe the nature of the rising clones that survived therapy. Technology limitation may cause underestimation of clonal diversity due to failure in identifying mutations clusters with MAF below 0.01, coverage bias in WXS, non-coding mutations, low tumour purity (for example, PARPNM relapse and PASLZM diagnosis) or subclonal SVs and CNVs that are below the detection level of SNP array and CGI WGS. Nevertheless, deep sequence coverage coupled with CNV and SV derived from SNP array assay and WGS enabled a comprehensive analysis of tumour clonal lineage from diagnosis to relapse for pediatric B-ALL at the genome-wide scale.

Our study is the first that compares the rising and falling clones to gain insight into the genetic and population characteristics of these differentially behaving subclones. Seventy-five per cent of the relapsed tumours are descendants of minor subclones at diagnosis, suggesting that in most cases the predominant clones were eradicated by therapy. Our study included cases that relapsed within 3 years of initial diagnosis, and the elevated *NT5C2* mutation rate in this cohort, 45% compared with the 3–10% by prior studies, is consistent with the signficant association of *NT5C2* mutation status with early relapse reported previously^6^,^7^. Of the 14 relapse-specific NT5C2 mutations identified in the nine cases, none were found in hypermutable tumours, indicating that hypermutation may represent an alternative mechanism for relapse. Notably, nine NT5C2 mutations from four cases (PAPJIB, PAPSPG, PAPZNK and PARFTR, Fig. 6) were in subclones descended from the relapse founder clones that had already acquired other relapse-specific mutations. For example, the two NT5C2 relapse-specific mutations (p.Arg367Gln and p.Ser360Pro) in PAPZNK were present in the two subclones evolved from a founder clone that has already gained eight relapse-specific mutations including mutations in two relapse-enriched genes *NRAS*, *USH2A* and a frameshift mutation in *WT1*, a known tumour suppressor gene (Supplementary Fig. 13). More strikingly, the PARFTR relapse specimen had four distinct NT5C2 mutations (p.Arg39Gln, p.Arg238Trp, p.Arg238Gln, and p.Arg367Gln) present in the four minor subclones that were descendants of the rising clone with WHSC1 p.Glu1099Lys mutation. The rising clone was present in 3% of the tumour cells at diagnosis (Supplementary Fig. 18). WHSC1, a gene enriched for relapse-specific mutations, had all four mutations in the SET domain including three with the recurrent p.Glu1099Lys oncogenic mutations known to promote leukaemic cell growth and self-renewal^16^. We previously reported ~40% of WHSC1 mutations in ALL to be subclonal^16^. Here we extend these findings by demonstrating WHSC1 mutated subclones at diagnosis to emerge as the founder clone at relapse.

The matched non-tumour samples used in this study were remission DNA obtained at day 28 following commencement of therapy, which may contain low levels of minimal residual disease^17^. Combining the high-coverage WXS and validation data, we were able to detect the presence of mutant alleles of 83 SNVs shared between diagnosis and relapse samples from 19 cases in these remission samples (Supplementary Data 6), raising the possibility of applying next-generation sequencing for disease monitoring which has been implemented for antigen-receptor genes^18^. For example, in remission DNA of PAPSPN, we found that JAK2 p.Arg683Gly, along with six other mutations in the founder clone of relapse, was present at MAFs of 0.001 to 0.005 (Supplementary Data 7). Further studies of patients with B-ALL using sensitive mutation testing at multiple time points during therapy are needed to determine if the persistence of low levels of the mutant clone at end induction might predict risk of relapse, and to examine the relationship between the nature of the therapeutic regimen used and the mutational spectrum.

These findings have major implications for understanding the genomic complexity of ALL. Construction of clonal lineage reveals the chronology of mutation acquisition in six major pathways, four of which were identified by our prior studies^14^. We have identified new targets of mutation at relapse, including *USH2A*, whose role in resistance to therapy is currently unknown and requires future mechanistic interrogation. This work highlights that the predominant clone present at diagnosis is eradicated by chemotherapy in the majority of cases, but with mutational turnover resulting in preservation of disruption in key pathways including Ras, JAK-STAT, lymphoid development and tumour suppressors throughout therapy. Thus, subclonal mutations affecting these key pathways should not be construed as being passenger mutations. In contrast, our findings of multiple subclonal mutations in the same gene or pathway suggest a different paradigm, in which such mutations are acquired in different cells after the earliest initiating event (often an oncogenic translocation or intrachromosomal rearrangement that creates a fusion gene)^19^,^20^ but contribute to the establishment of multiple fully leukemogenic subclones at diagnosis. For example, our clonal lineage analysis shows that nearly all mutations in the Ras signalling pathway at diagnosis were acquired after emergence of founder clone. Additional mutations are acquired that determine fitness in the face of anti-leukaemic therapy and determine which of these mutations are extinguished or survive to seed relapse.

Our findings also have direct implications for the clinical management of ALL. Several mutations confer resistance to specific therapeutic agents, including nucleoside analogues (*NT5C2*) and glucocorticoids (*CREBBP*)^5^, thus alternative treatment approaches to avoid mutation selection or mitigate resistance may be needed. The high frequency of mutations in epigenetic regulators provides further support that agents targeting epigenetic modifications represent a new therapeutic opportunity in ALL^21^. Our findings also suggest that there may be clinical utility to assaying for specific mutations that might be a harbinger of relapse during the course of ALL treatment. While this strategy is widely applied using antigen receptor gene rearrangements, those are non-oncogenic events present in both fully leukaemic and ancestral preleukaemic cells. Sensitive mutation detection strategies focused on known oncogenic driver mutations could complement existing methods to detect low levels of minimal residual disease and warrant further investigation.

## Methods

### Patients and samples

All patients or their parent(s)/guardian(s) provided consent for the collection and banking of diagnostic and remission specimens. The study was approved by the St Jude Children’s Research Hospital Institutional Review Board.

### Summary of data analysis

The diagnosis–remission–relapse DNA samples were obtained from 20 patients treated on recent COG B-ALL trials. Exome sequencing, WGS and SNP array data were performed using standard Illumina, Complete Genomics and Affymetrix protocols followed by data analysis, including WXS mapping, coverage and quality assessment, SNV/indel detection, tier annotation for sequence mutations, is described below. Clonal lineage was derived from mutation clusters constructed by a binomial mixture model (see below) that explicitly accounts for read coverage and determines mutation clusters that are diagnosis specific, relapse specific and common. CNV and SV were incorporated into the estimated lineages when appropriate.

For each sample, the MAFs of coding SNVs (including both silent and non-silent) in diploid regions were subjected to an unsupervised clustering analysis by R package mclust. The tumour purity was estimated by twice the highest cluster center value among all cluster centres ≤0.5. Diagnosis and relapse samples were examined separately.

### Binomial mixture model

All experimentally verified somatic SNVs located in diploid chromosomal regions were included in this analysis. Diagnosis (D)-specific or relapse (R)-specific SNVs were analysed separately from those that were shared between D and R. D- or R-specific SNVs were represented as (*X*_*i*_,*N*_*i*_),*i*=1,2,…,*n*, where *X*_*i*_ and *N*_*i*_ denote the counts of mutant allele reads and total reads for *i*-th SNV, respectively. Here MAF (mutant allele frequency) *p*_*i*_ for *i*-th SNV is estimated to be 
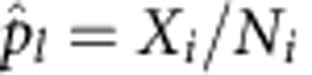
 and mutant allele count is modelled by binomial distribution *X*_*i*_~*Binom*(*p*_*i*_,*N*_*i*_). To determine the clonal origin of each SNV, subclones are denoted as *C*_*k*_, with corresponding population fraction *p*_*k*_, for *k*=1,2,…,*K* and subclonality membership is determined by function *Z*_*ik*_, which takes value 1 when *i*-th SNV belongs to subclone *C*_*k*_ and 0 otherwise. The probability distribution of *X*_*i*_ can be rewritten as




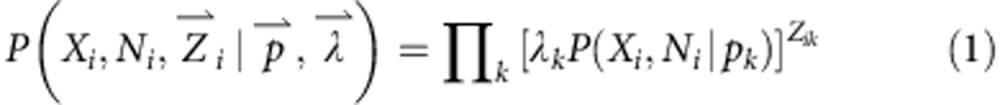




where 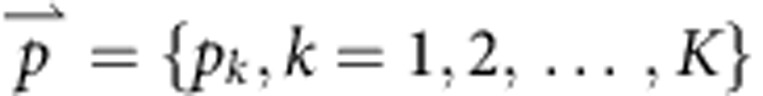 and λ_*k*_ is the mixing parameter.

In turn, the joint probability distribution of data {(*X*_*i*_,*N*_*i*_)} can be written as




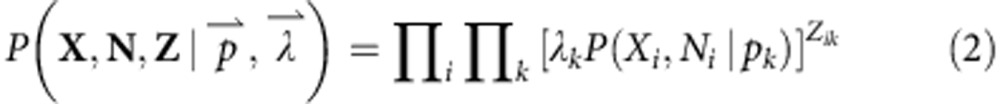




where **X**={*X*_*i*_,*i*=1,2,…,*n*}, **N**={*N*_*i*_,*i*=1,2,…,*n*} and **Z**={*Z*_*ik*_,*i*=1,2,…,*n*; *k*=1,2,…,*K*}.

The likelihood of model parameters 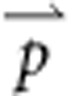 and 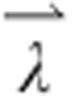 given **X** and **Z** is defined as




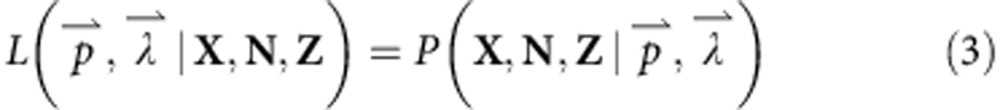




The above likelihood is maximized using expectation maximization algorithm with Akaike information criterion (AIC) AIC=2*k*−2ln(*L*), where *k*=(*n*+2)−1.

For shared SNVs present in both D and R, the above binomial mixture model was modified to a multiplicative binomial mixture model as




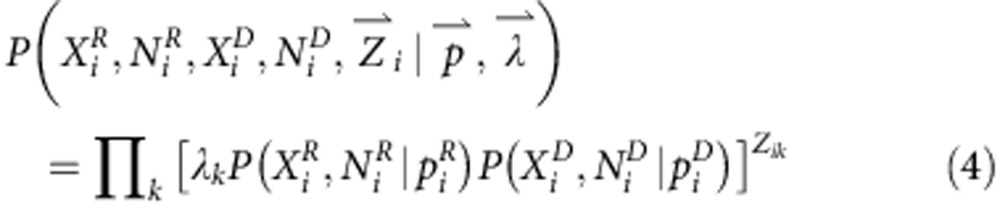




where 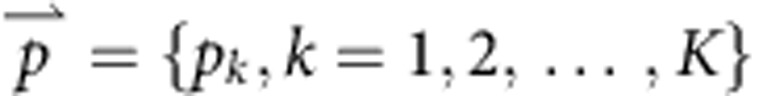 and λ_*k*_ is mixing parameter.

The joint probability distribution of data 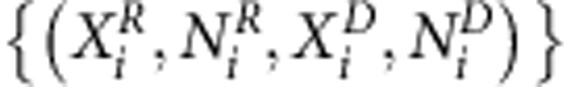 is written as




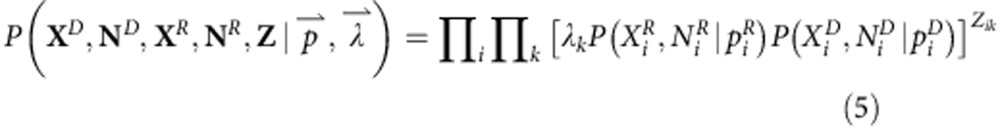




The likelihood of model parameters 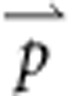 and 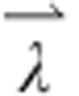 given **X**, **N** and **Z** is defined as




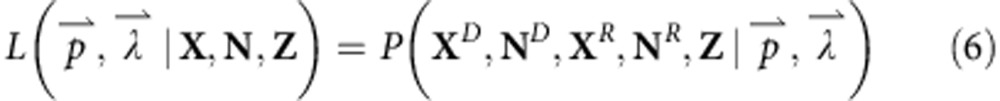




where **X**, **N** and **Z** are defined similarly as above.

The above likelihood is maximized using an expectation maximization algorithm with the AIC=2*k*–2ln(*L*), where *k*=(*n*+3)*K*–1.

Each mutational cluster inferred from the binomial mixture models was labelled by genes involved in a significantly mutated pathway (Fig. 1) or a somatic variant with the highest coverage if there is no mutation in the selected pathway.

### Clonal lineage inference

Clonal lineage was inferred from an approach applied in prior studies^8^ with several enhancements. Specifically, clonal lineages were constructed separatly for mutation clusters categorized as D-specific, R-specific and shared SNVs. The cluster of shared SNVs were analysed first and the cluster that had the highest MAF at diagnosis is comprised of genetic lesions of the founder clone, that is, the ancestral clone for all subclones identified at diagnosis and relapse. Starting from the founder clone at diagnosis, we first built lineages for the shared SNV clusters at diagnosis, in descending order of their population size estimated from MAF. Specifically, for each lineage *J*, denote the set of shared mutational clusters with established clonal lineages as {*C*_*j*_,*j*=1,2,…*J*}, each with a population size {*F*_*j*_,*j*=1,2,…*J*}, *J*=1 refers to the founder/ancestral clone. For a new shared mutational cluster *C*_*J*+1_, we determined its parental clone as subclone *C*_*j*0_ with the highest population size *F*_*j*0_>*F*_*k*_, for *k*≠*j*0. In turn, the population size for subclone *C*_*j*0_ is adjusted to *F*_*j*0_–*F*_*J*+1_. This process was iterated until all shared mutational clusters at diagnosis were assigned to a parental clone. The D-specific clusters were subsequently added to this lineage tree with the same logic. The clonal lineage is determined similarly for the R-specific clusters. The founder clones at relapse are mapped to subclones at diagnosis based on shared mutational clusters. We allowed multiple progenitor clones in relapse in this mapping as the relapsed tumour could consist of mutations present in multiple subclones at diagnosis. An example can be found in case PARJZZ (Fig. 4).

Clonal lineages were manually reviewed to identify those with potential alternative parental lineages other than the default assigned by the algorithm (that is, the clone with the highest population size). For cases with alternative clonal lineage assignment, we attempted to resolve lineage ambiguity by incorporating biological knowledge. Specifically, a lineage that is consistent with mutual exclusivity of activating mutations in the same gene or the same pathway or a parental clone that exhibits consistent mutation spectrum will be selected preferentially. This was used to resolve clonal ambiguity of PAPSPN, PASLZM, PAPNNX (partial), PAPLDL, PAPZNK, PAPJIB, PARGHW, PARFTR and PARPNM. When biological knowledge is unavailable, unresolved ambiguous lineages are highlighted in the lineage graphs and alternative lineage graphs are provided.

### Clonality of SVs and CNVs

SVs and CNVs affecting the genes involved in the six key pathways as well as the known oncogenic fusions were included for clonal lineage construction. Specifically, SVs with breakpoints disrupting the genes of interest and CNVs spanning ≤5 genes were included. Because of the limitation in detecting subclonal SVs (due to read-depth and mapping bias in WGS) and CNVs (due to resolution of SNP array), clonality of SVs/CNVs was assessed qualitatively, that is, whether they are shared, diagnosis specific or relapse specific. Since oncogenic fusions are likely the initiating events of childhood ALL^19^,^20^, SVs/CNVs were considered to be present in the predominant clone (or the ancestral clone) by default with two exceptions: (1) SVs detected by deep sequencing of the trio samples by capture sequencing but not by WGS which indicates presence of subclonal SV at diagnosis (for example, the *CREBBP* SV identified in case PARBRK); and (2) CNVs classified as relapse specific by automated analysis but were found to be subclonal at diagnosis by manual review of raw probe intensity of SNP array. SVs and CNVs were placed into the clonal lineage constructed from MAF of sequence mutations unless insertion of a new branch was required to account for their presence (for example, PAPJIB).

### Whole-exome sequencing

DNA samples were constructed into Illumina paired-end libraries and processed through exome capture according to the manufacturer’s specifications with modifications as described in the BCM-HGSC Illumina Non-Barcoded Paired-End Capture Library Preparation protocol ( https://www.hgsc.bcm.edu/sites/default/files/documents/BCM-HGSC_Illumina_Non-Barcoded_Paired-End_Capture_Library_Preparation.pdf).

‘Precapture libraries’ were prepared using robotic workstations (Biomek NXp and FXp models, Beckman Coulter Genomics). In brief, 1 or 5 μg of DNA was sheared into fragments of ~400 bp in a plate using a E210 system (Covaris Inc., Woburn, MA) followed by end-repair, A-tailing and ligation of non-barcoded PE adaptors (Illumina, San Diego, CA). Precapture Ligation Mediated-PCR (LM-PCR) was performed for seven cycles of amplification using the 2 × SOLiD Library High Fidelity Amplification Mix (a custom product manufactured by Invitrogen Inc.). Purification was performed with Agencourt AMPure XP (Beckman Coulter Inc.) beads after enzymatic reactions. Following the final XP beads purification, quantification and size distribution of the precapture LM-PCR product was determined using the LabChip GX electrophoresis system (PerkinElmer).

For exome capture, precapture libraries (~1 μg) were individually hybridized in solution to the SeqCap EZ Exome 2.0 design (44 Mb, NimbleGen). Human Cot1 DNA and 3′-ddC-modified hybridization enhancing oligonucleotides were added into the hybridization to block repetitive genomic sequences and the adaptor sequences. Post-capture LM-PCR amplification was performed using the 2 × SOLiD Library High Fidelity Amplification Mix with 14 cycles of amplification. After the final AMPure XP bead purification, quantity and size of the capture library was analysed using the Agilent Bioanalyzer 2100 DNA Chip 7500. The efficiency of the capture was evaluated by performing a quantitative PCR-based quality check on the four standard NimbleGen internal controls. Successful enrichment of the capture libraries was estimated to range from a 6 to 9 of Δ*C*t value over the non-enriched samples.

Library templates were prepared for sequencing using Illumina’s cBot cluster generation system with TruSeq PE Cluster Generation Kits (Part no. PE-401–2001). In brief, these libraries were denatured with sodium hydroxide and diluted to 3–6 pM in hybridization buffer to achieve a load density of ~800 K clusters mm^−2^. Each library was loaded in a single lane of a HiSeq flow cell, and each lane was spiked with 2% phiX control library for run quality control. The sample libraries then underwent bridge amplification to form clonal clusters, followed by hybridization with the sequencing primer. Sequencing runs were performed in paired-end mode using the Illumina HiSeq 2000 platform. Using the TruSeq SBS Kits (Part no. FC-401-1001), sequencing-by-synthesis reactions were extended for 101 cycles from each end. Sequencing runs generated ~150–250 million successful reads on each lane of a flow cell, with an average of 20 Gb per sample. With these sequencing yields, samples achieved an average of 90% of the targeted exome bases covered to a depth of 20 × or greater.

### Whole-genome sequencing

WGS for 16 cases was performed by Complete Genomics. Details on sequencing, data analysis and coverage are available at ftp://caftpd.nci.nih.gov/pub/dcc_target/ALL/Phase_II/sequence/WGS/CGI_TARGET_Pipeline_README.pdf.

### Data analysis

Initial sequence analysis was performed using the HGSC Mercury analysis pipeline ( https://www.hgsc.bcm.edu/software/mercury). First, the primary analysis software on the instrument produced .bcl files that were transferred off-instrument into the HGSC analysis infrastructure by the HiSeq Real-time Analysis module. Once the run was complete and all .bcl files were transferred, Mercury ran Illumina’s primary analysis software (CASAVA), which demultiplexes pooled samples and generates sequence reads and base-call confidence values (qualities). This was followed by mapping of reads to the GRCh37 human reference genome ( http://www.ncbi.nlm.nih.gov/projects/genome/assembly/grc/human/) using the Burrows-Wheeler aligner (BWA^22^, http://bio-bwa.sourceforge.net/) and producing a BAM^23^ (binary alignment/map) file. The third step involves quality recalibration (using GATK^24^, http://www.broadinstitute.org/gatk/), and where necessary the merging of separate sequence-event BAMs into a single sample-level BAM. BAM sorting, duplicate read marking and realignment to improve indel discovery all occur at this step.

A second phase of data analysis was performed at St Jude Children’s Research Hospital for detecting somatic SNVs and insertion/deletion (indels) with an established pipeline^4^,^5^. In brief, the reference human genome assembly GRCh37-lite was used for mapping all samples. The coverage of each tumour was summarized in Supplementary Fig. 1. Putative SNVs/indels were called using Bambino^6^, and potential false positive variants caused by alignment artifacts, poor quality, and paralogs were filtered. SVs were predicted from WXS data using the CREST algorithm^7^. All SNVs, indels and SVs affecting gene coding regions were submitted for capture validation.

Affymetrix SNP 6.0 array data analysis was performed by incorporating optimal reference normalization^9^ and circular binary segmentation^10^. The results are shown in Supplementary Data 4. All putative CNVs identified by this pipeline were manually reviewed in dChip and only focal CNVs (≤5 genes) and CNVs involving one of *CDKN2A*, *PAX5* and *TCF3* were incorporated in the pathway analysis.

SVs predicted by analysis of complete genomics WGS data were submitted to the SV annotation pipeline FusionBuilder as previously described^4^,^5^. SVs resulting in in-frame gene fusions or with support from CNVs determined by SNP array were submitted for capture validation.

### Experimental verification

All sequence mutations (including SNVs and indels) and a subset of SVs selected from WXS and CGI WGS analysis (with selection criteria described in the data analysis section) were subjected to experimental verification. An exception was made for two hypermutators with >300 relapse-specific mutations, PAPNNX and PASFXA: ~100 SNVs were selected for verification for each case. The targeted regions were submitted for probe design to Nimblegen/Roche using Nimbledesign V1.2. Library construction was performed using the NextFlex DNA Sequencing library prep kit (Bioo Scientific, Austin, TX). Targeted enrichment was conducted according to manufacturer’s recommendations (Roche), NGS sequencing using the PE100 protocol with V3 reagents was performed on a HiSeq 2000. The overall verification rate was 98%. The verified variants and high-quality putative variants not subjected for verification are listed in Supplementary Data 2 and were used for clonal evolution analysis. The verification for 24 SNVs was supplemented by amplicon NGS using a MiSeq (PE 150 cycle protocol). The PCR amplification was accomplished using Amplitaq Gold 360 (Life Technologies). The Nextera XT library construction kit (Illumina) was used to generate NGS libraries from the amplicons.

### Site-specific screening of low MAF variants

To account for low MAF variants that are below the threshold of our SNV/indel detection pipeline, we employed a site-specific screening of each validated SNV or indel for presence of mutant allele in any of the diagnosis–relapse–remission trio samples with a negative mutation call. NGS reads harbouring the mutant allele based on the alignment stored in bam file were extracted for further analysis to determine the validity of the mutant allele. Each read was realigned using the Smith–Waterman algorithm to the hg19 reference genome including 100-bp flanking sequence around the variant. If the mutant allele was absent after realignment, the read was considered to be wild-type, and the mutant allele a false positive resulting from alignment artefact generated by the mapping algorithm. For a read that survived this alignment check, we performed the following check to filter variant caused by sequencing error or PCR artefact based on the evaluation of verification assay: (a) location of the mutant allele represents the lowest point (‘valley’) of sequence quality in a 5-bp window; or (b) the mutant allele was present only in sequence fragments with overlapping forward and reverse reads. The final mutant allele count included only the NGS reads that passed both the alignment check and variant allele quality check. We required a minimum of three non-duplicate reads to determine the presence or absence of a mutant allele at diagnosis or relapse using the combined data from WXS and capture sequencing.

### Power for detecting low-frequency mutations

The mean coverage for WXS and capture sequencing were 187- and 696-fold, respectively. As capture sequencing was performed on DNAs derived from diagnosis, relapse and remission trio, we were able to recover low-frequency mutations at diagnosis missed by WXS. The combined coverage of WXS and capture sequencing is 883-fold, which was used to calculate MAF for clonal evolution analysis.

Using a binomial distribution, the power for detecting mutation at a MAF of 0.01 was 28.8, 97, 99.3%, respectively, for WXS, capture-alone and combined data. The power for discovering mutations at MAF of 0.03 and 0.05 by WXS is 92.13 and 99.6%, respectively.

### Clonal origin of mutations in relapsed tumour of cases PAPSPN and PASLZM

The rising subclone (clone 3 in Fig. 3b) had a mean MAF of 0.003 (considering *JAK2* p.Arg683Gly and *KRAS* p.Ala146Thr), raising the possibility that relapse-specific mutations (cluster *e* in Fig. 3a) representing the founding clone of relapse (clone 4 in Fig. 3b) should be part of clone 3 but were not detected at diagnosis due to low frequency. To address this question, we used the MAF (denoted *α*=0.003) of clone 3 at diagnosis, the combined diagnosis tumour coverage (denoted N) to calculate the probability of misclassifying each of the 38 SNVs present in cluster *e* as P(*X*=0|*N*, *α*) using a binomial distribution. A SNV with probability ≤0.05 was unlikely to be misclassified. A compound *P* value is then calculated as *P*(*X*>=*x*|#SNV, 0.05) using binomial distribution where *x* is the number of SNVs that are called unlikely to be misclassified and #SNV is the total number of cluster *e* SNVs, and 0.05 is the cutoff to call an SNV unlikely to be misclassified. This analysis showed that 14 out of the 38 cluster *e* SNVs had *P* value<0.05, which resulted in a compound *P* value 2e−9, suggesting that the mutation cluster *e* (clone 4) as a whole was unlikely to be the same as mutation cluster *d*.

Using a similar approach, we evaluated the clonal original of mutation clusters present in the dominant clone in the relapsed tumour of case PASLZM. Clone 2, defined by its lineage-specific mutation cluster *b*, is the rising subclone that persisted from diagnosis to relapse. The MAF of cluster *b* is ~0.01 at diagnosis and 0.5 at relapse. Mutation cluster *c* is comprised of five SNVs and their mutant allele is clonal at relapse but absent at diagnosis. Combining the coverage of WXS and capture data, the minimum coverage for each SNV in cluster *c* is 520. Under the assumption that cluster *c* were part of cluster *b* with a MAF of 0.01 at diagnosis, the binomial *P* value for detecting 0 mutant allele for each SNV is 0.003, while the compound *P* value for detecting 0 read for all five SNVs is 3e−7. This result suggests that mutation cluster *c* is likely to be distinct from cluster *b*.

## Additional information

**Accession codes.** The sequence data of B-progenitor ALL samples have been deposited in dbGAP ( http://www.ncbi.nlm.nih.gov/gap) under the accession code phs000218.

**How to cite this article:** Ma, X. *et al.* Rise and fall of subclones from diagnosis to relapse in pediatric B-acute lymphoblastic leukaemia. *Nat. Commun.* 6:6604 doi: 10.1038/ncomms7604 (2015).

## Supplementary Material

Supplementary Information Supplementary Figures 1-23 and Supplementary References.

Supplementary Data 1 Clinical information of all 20 trios in this study

Supplementary Data 2 SNVs and Indels of all 20 trios in this study.

Supplementary Data 3 Structural Variations in all 20 trios in this study

Supplementary Data 4 Copy number variations detected by SNP6.0 array.

Supplementary Data 5 Ten tumors that have multi-subclonal mutations in the same gene.

Supplementary Data 6 Mutant alleles in remission DNA.

Supplementary Data 7 Detection of mutant allele in remission DNA in case PAPSPN.
