# Supplementary material for: Rise and fall of subclones from diagnosis to relapse in pediatric B-acute lymphoblastic leukaemia
Source: Nat Commun. 2015 Mar 19;6:6604. doi: 10.1038/ncomms7604 (PMC4377644; doi:10.1038/ncomms7604)
Supplement: Supplementary Information — Supplementary Figures 1-23 and Supplementary References. [file ncomms7604-s1.pdf]

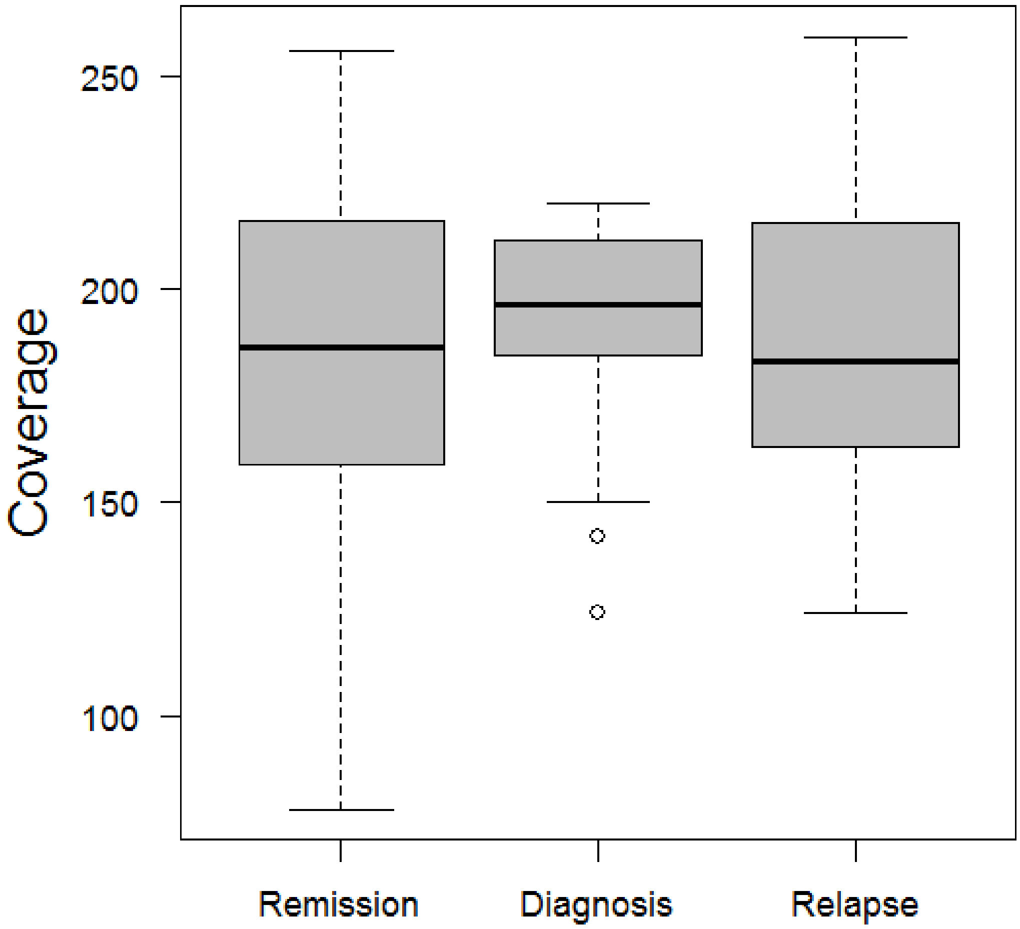

Supplementary Figure 1. Box plot of median coverage of coding exons of the 20 cases analyzed by whole-exome sequencing.

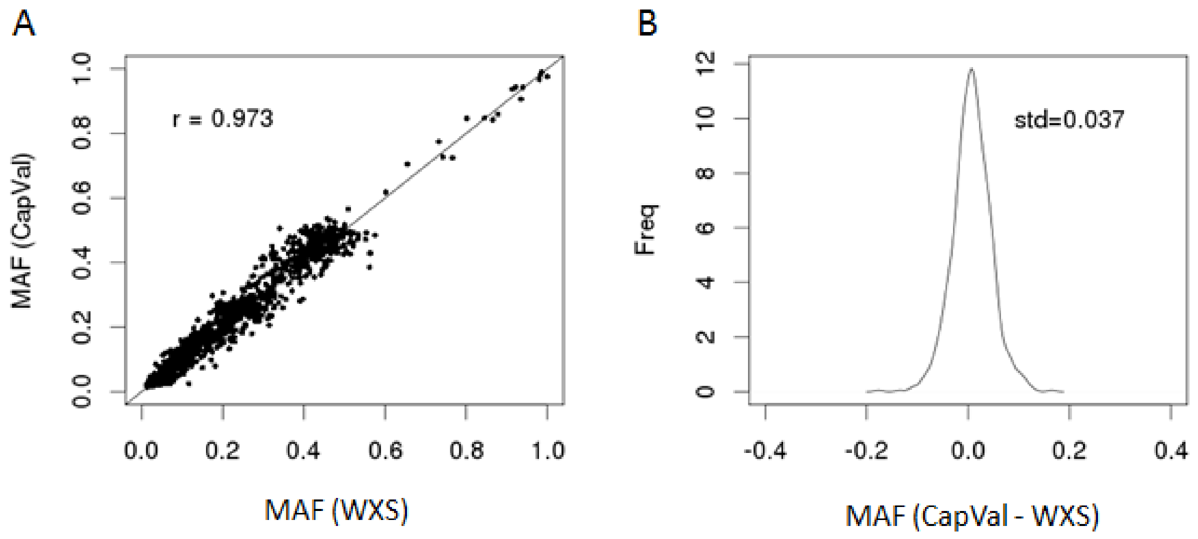

Supplementary Figure 2. Concordance of Mutant Allele Fraction between WXS and capture validation (CapVal).

A) Comparison of raw MAF between WXS and CapVal (Pearson's correlation coefficient r=0.973). B) Density plot of MAF difference between CapVal and WXS. The mean difference is at 0.007. Only SNVs with >100x coverage from WXS are included.

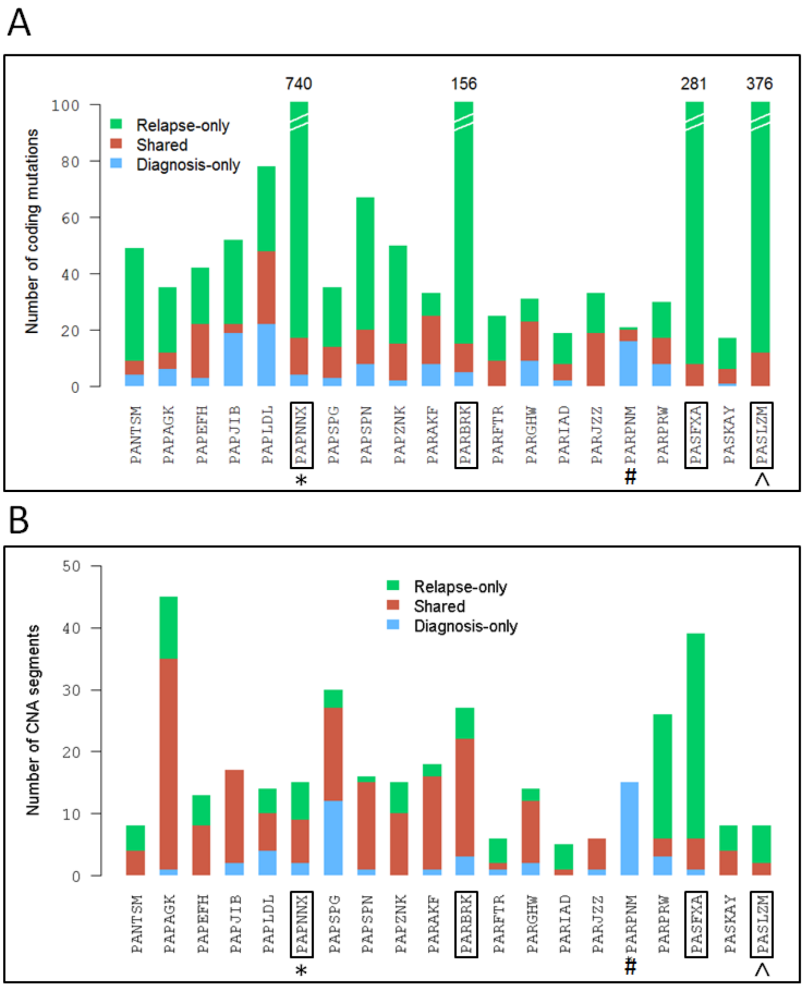

Supplementary Figure 3. Comparison of number of somatic lesions identified in diagnosis and relapse.

Diagnosis-specific lesions, i.e. the lesions that did not persist to relapse, are marked in blue. Lesions found at both diagnosis and relapse, i.e. lesions at diagnosis that persisted to relapse, are marked in red. Relapse-specific lesions, i.e. lesions that were acquired at relapse, are marked in green. The sample marked with *, PAPNNX, has relapse-specific mutations in *MSH6* and *MLH1*. The sample marked with ^, PASLZM, has a relapse-specific *PMS2* splice-site mutation. Case PARPNM has low tumor purity in relapse sample (<20%) and is marked with a #. Four cases (PAPNNX, PASFXA, PASLZM, PARBRK) that have high mutation rate in relapse are considered hypermutable tumors and marked in box. A) Comparison of coding mutations including SNVs and indels. The y-axis is capped at 100 mutations per case. B) Comparison of copy number alterations in diagnosis and relapse.

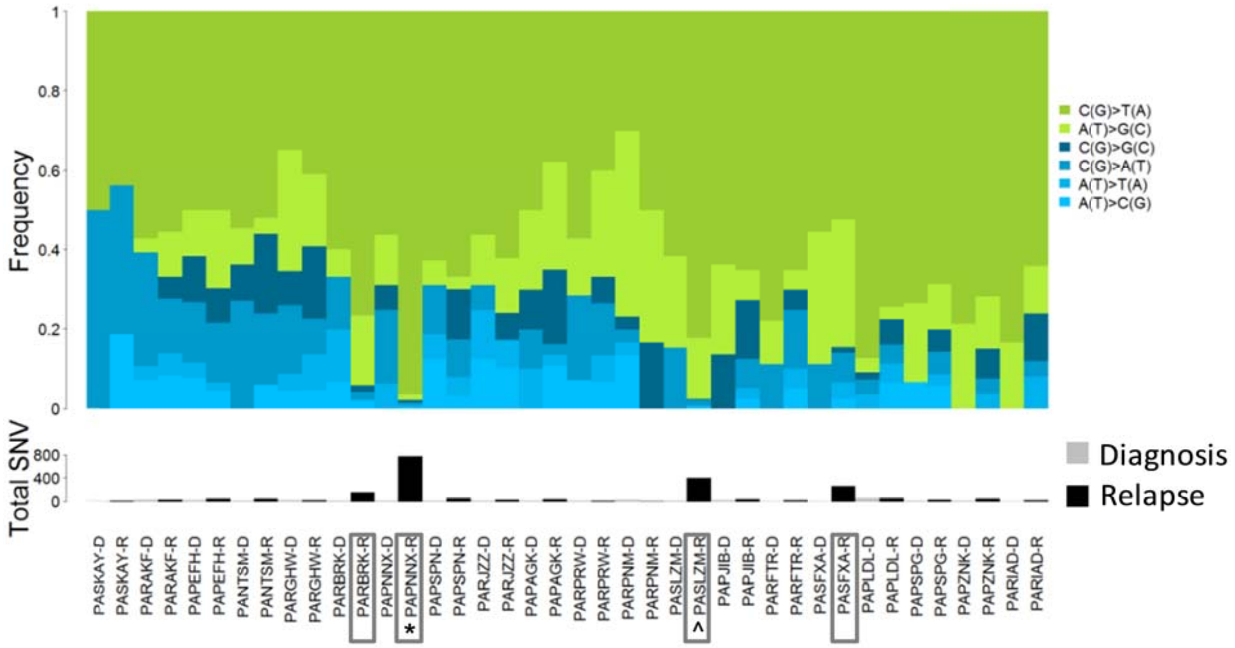

Supplementary Figure 4. Mutation Spectrum in Diagnosis vs Relapse.

Tumors are displayed in diagnosis (-D) and relapse (-R) pairs. Number of somatic SNVs identified in each tumor is shown at the bottom panel. The transition mutations are marked in green while the transversion mutations are shown in blue. Hypermutable relapsed tumors and their mutation status in DNA mismatch genes are marked the same as in Supplementary Figure 3.

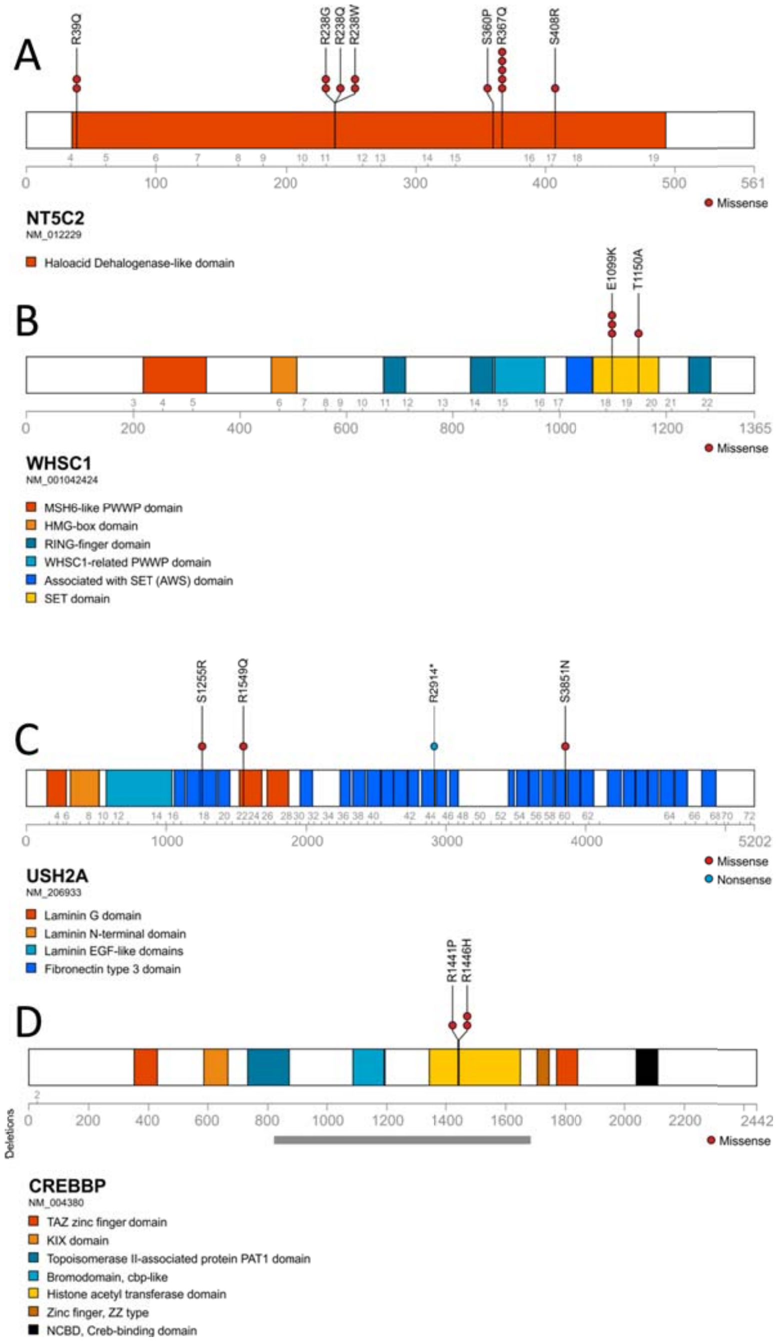

Supplementary Figure 5. Non-silent mutations in four genes that are enriched for relapse-specific mutations.

A) *NT5C2*, all mutations are relapse-specific. B) *WHSC1*, 3 out of 4 mutations are relapse-specific; C) *USH2A*, 3 out of 4 mutations are relapse-specific; and D) *CREBBP*, all sequence mutations are relapse-specific while a focal deletion that removes amino acid 822-1682 was subclonal at diagnosis and was present in the predominant clone at relapse in case PARBRK.

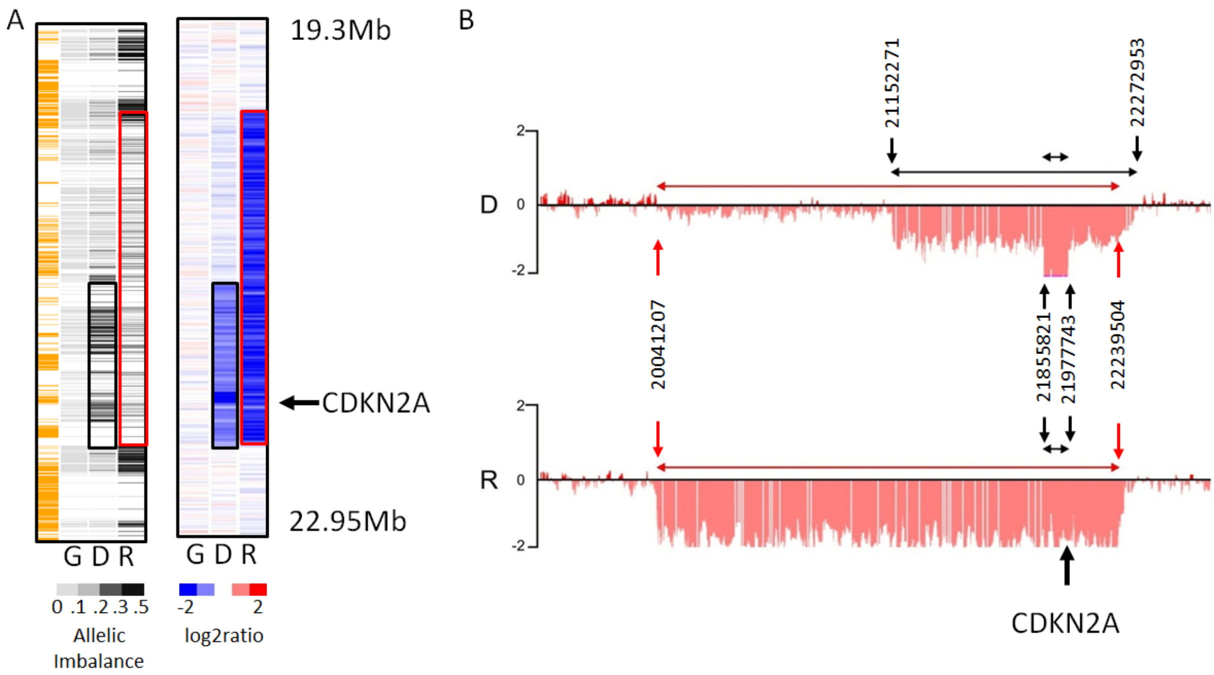

Supplementary Figure 6. Multi-clonal CDKN2A deletion in patient PARJZZ.
(A) LOH (left panel) and CNV (right panel) on chr9:19.3-22.95Mb in remission DNA (marked as G), diagnosis (marked as D) and relapse (marked as R) of case PARJZZ. The location of *CDKN2A* is marked by an arrow. LOH is inferred from allelic imbalance, i.e. ABS|$MAF_{non\_ref\_allele}$-0.5|, and is shown in gray-black scale. High allelic imbalance (dark color) at D or R but low in G (light color) represents regions of loss of heterozygosity. Only germline SNVs from CGI WGS data that have MAF in [0.4, 0.6] with coverage >20x in G and >5X in D and R samples (to account for focal deletions) are included. The leftmost orange track displays SNVs with MAF>0.9 in G sample, indicating regions of homozygosity in germline that account for gaps in LOH track. The CNV profile (right) marks the log_2ratio of each probe on the SNP6.0 array. The black and red boxes mark the predominant copy number loss found at diagnosis (black) and relapse (red) and this color code also matches the *CDKN2A* deletions in the clonal evolutionary graph of PARJZZ in Fig. 4. At diagnosis, two independent deletion events resulted in homozygous deletion of *CDKN2A*. At relapse, the predominant homozygous deletion is flanked by copy-neutral LOH, suggesting that the tumor first acquired a heterozygous deletion followed by loss of heterozygosity. Its boundary matches the light blue track detectable manually at diagnosis. B) Probe intensity at *CDKN2A* locus presented as wiggle plot of log_2ratio. Horizontal arrows mark the predominant CNVs detected at diagnosis (black color) and relapse (red color). Vertical arrows mark the genomic coordinates of SVs detected at diagnosis (black) and relapse (red) by CGI WGS. Both WGS SV and SNP array probe intensity shows that the large homozygous deletion at relapse is subclonal at diagnosis. The predominant focal homozygous deletion of *CDKN2A* at diagnosis is present at subclonal level at relapse based on SV derived from CGI WGS.

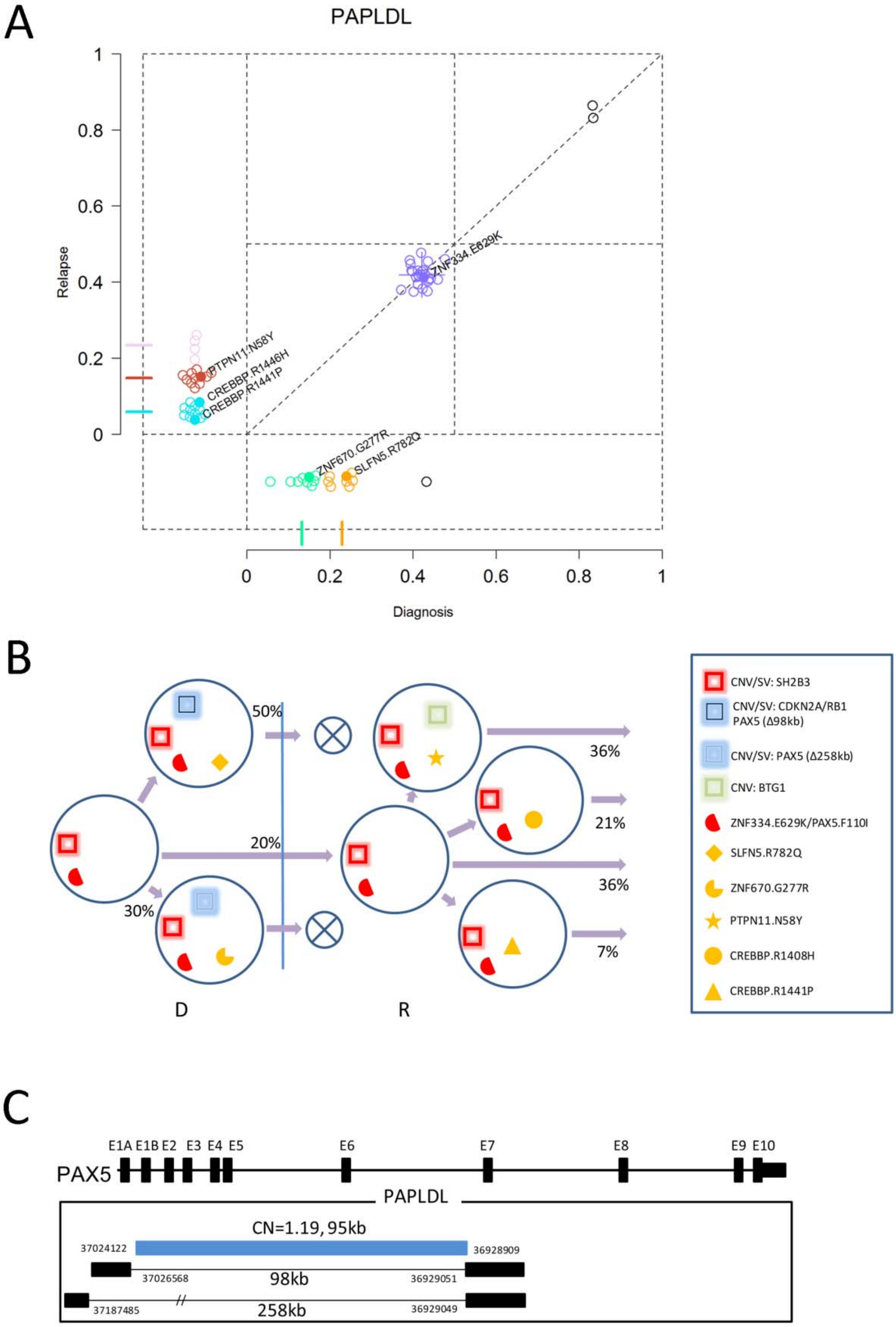
A
PAPLDL
Relapse
Diagnosis
0
0.2
0.4
0.6
0.8
1
PTPN11.N58Y
CREBBP.R1446H
CREBBP.R1441P
ZNF334.E629K
ZNF670.G277R
SLFN5.R782Q
B
50%
20%
30%
36%
21%
36%
7%
D
R
CNV/SV: SH2B3
CNV/SV: CDKN2A/RB1
PAX5 (Δ98kb)
CNV/SV: PAX5 (Δ258kb)
CNV: BTG1
ZNF334.E629K/PAX5.F110I
SLFN5.R782Q
ZNF670.G277R
PTPN11.N58Y
CREBBP.R1408H
CREBBP.R1441P
C
E1A E1B E2 E3 E4 E5
E6
E7
E8
E9 E10
PAX5
PAPLDL
CN=1.19, 95kb
37024122
36928909
37026568
98kb
36929051
37187485
258kb
36929049

Supplementary Figure 7. Clonal architecture of diagnostic (D) and relapsed (R) tumors for PAPLDL.

A) Scatter plot of mutant allele fraction (MAF) of diploid SNVs (i.e., no copy number changes from SNP6 array data indicated in Supplementary Data 4) in case PAPLDL. The relapse MAF is shown in y-axis and the diagnosis MAF is shown in x-axis. Refer to Figure 3 for display regions. SNVs are colored according to their cluster memberships determined by our binomial mixture model for shared, diagnosis-specific, and relapse-specific SNVs, respectively. Singleton outliers and SNVs with MAF>0.55 are colored black since they might be from copy-number-neutral LOH (CN-LOH) regions. Clonal MAF (i.e., cluster centers estimated by our binomial mixture models) are indicated with a short horizontal bar (for relapse specific SNVs) or a short vertical bar (for diagnosis specific SNVs) or a big "+" (for shared SNVs) with corresponding colors. Selected cancer driver genes (from Figure 1) and cluster label genes are indicated with solid dots. B) Clonal evolution of PAPLDL. Deletion of *SH2B3* was present in the founder clone at diagnosis. The founder clone had two descendant subclones, one with a 98 kb deletion involving exons 1B, 2-6 of *PAX5* and *CDKN2A*, *RB1* deletions, which accounted for 50% of the diagnosis tumor cells. The other had a 258 kb deletion involving exons 1A through 6 of *PAX5* gene, and it accounted for 30% of diagnosis tumor cells. Both descendant clones were eliminated by therapy resulting in absence of *PAX5* deletion and SVs in relapse. Both *PAX5* subclones were assigned as daughter subclones of the ancestral clone based on mutual exclusivity rule. The ancestral clone, which was present in 20% of the tumor cells at diagnosis, persisted to relapse and co-existed with its three descendant subclones. Two descendant subclones acquired distinct relapse-specific mutations in *CREBBP*: p.Arg1441Pro and p.Arg1408His and the third acquired a *PTPN11* p.Asn58Tyr mutation at relapse. We assigned all three subclones as sister subclones based on mutual exclusivity rule. C) Multiple focal deletions of *PAX5* in PAPLDL diagnosis sample. Gene model is illustrated in top panel with exons depicted by thick boxes, indexed by exon 1A through exon 10. Copy number changes detected by SNP6 array was indicated by a filled blue box. Structural variations were indicated by a thin line connecting two filled black boxes. Genomic coordinates of the events were indicated.

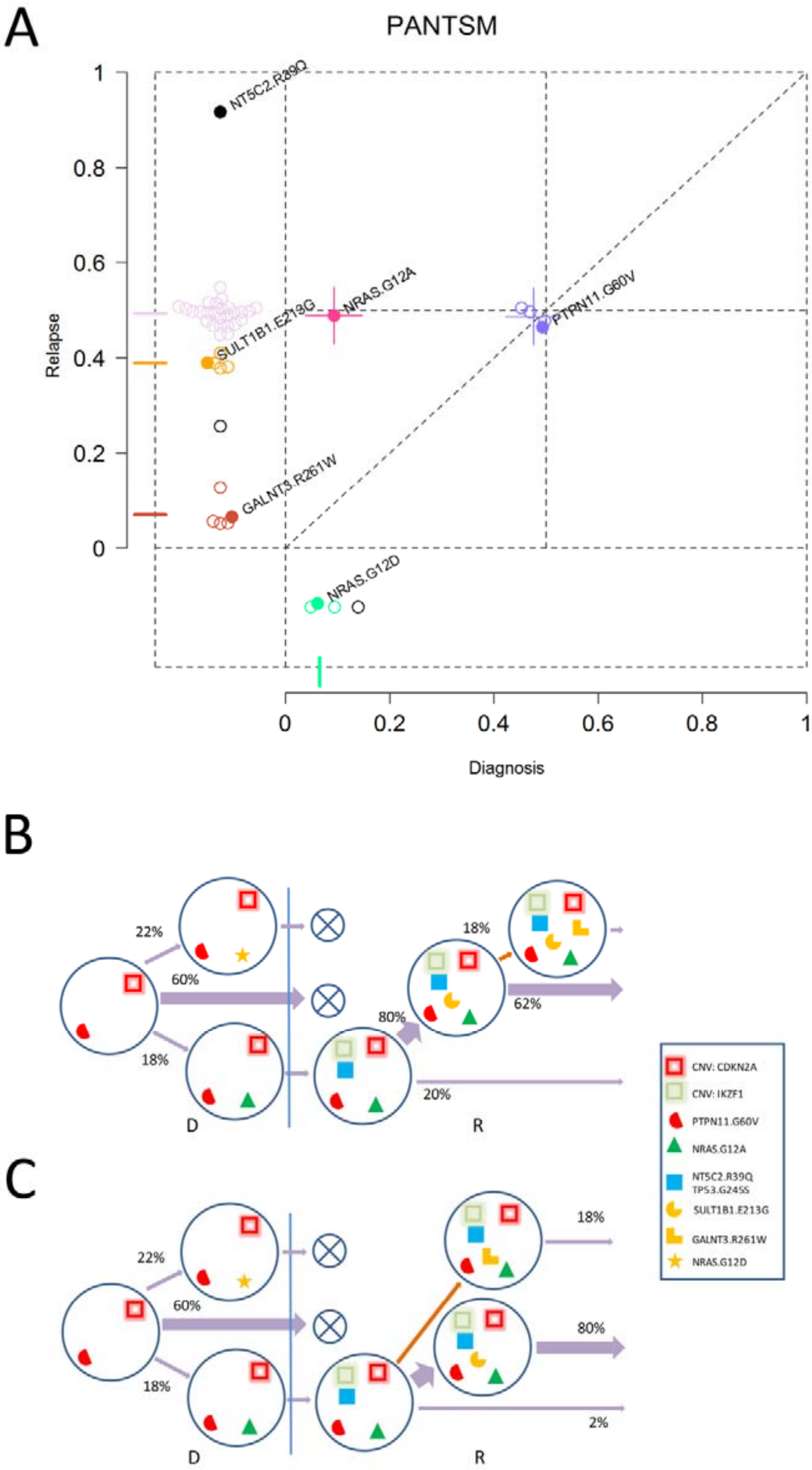
A
PANTSM
NT5C2.R39Q
NRAS.G12A
PTPN11.G60V
SULT1B1.E213G
GALNT3.R261W
NRAS.G12D
Relapse
Diagnosis
0
0.2
0.4
0.6
0.8
1
B
22%
60%
18%
80%
62%
20%
D
R
C
22%
60%
18%
18%
80%
2%
D
R
CNV: CDKN2A
CNV: IKZF1
PTPN11.G60V
NRAS.G12A
NT5C2.R39Q
TP53.G245S
SULT1B1.E213G
GALNT3.R261W
NRAS.G12D

Supplementary Figure 8. Clonal architecture of diagnostic (D) and relapsed (R) tumors for PANTSM.

A) Scatter plot of mutant allele fraction (MAF) of diploid SNVs. B) Loss of *CDKN2A* is present in the ancestral founder clone at diagnosis along with *PTPN11* p.Gly60Val mutation. There are two subclones harboring distinct *NRAS* mutations at diagnosis: one had *NRAS* p.Gly12Asp and was estimated to account for 22% of the tumor cells, and was eliminated by therapy. The other had *NRAS* p.Gly12Ala. This subclone accounted for 18% of the tumor cells at diagnosis and persisted to become the founder clone of relapse after acquiring several relapse-specific mutations including *NT5C2* p.Arg39Gln, *TP53* p.Gly245Ser and an *IKZF1* deletion. It also has two descendant subclones at relapse. The orange arrow at the relapse indicates an alternative lineage of the subclone with the *GALNT3* mutation as shown in C). Scenario B) is used for Figure 6.

A

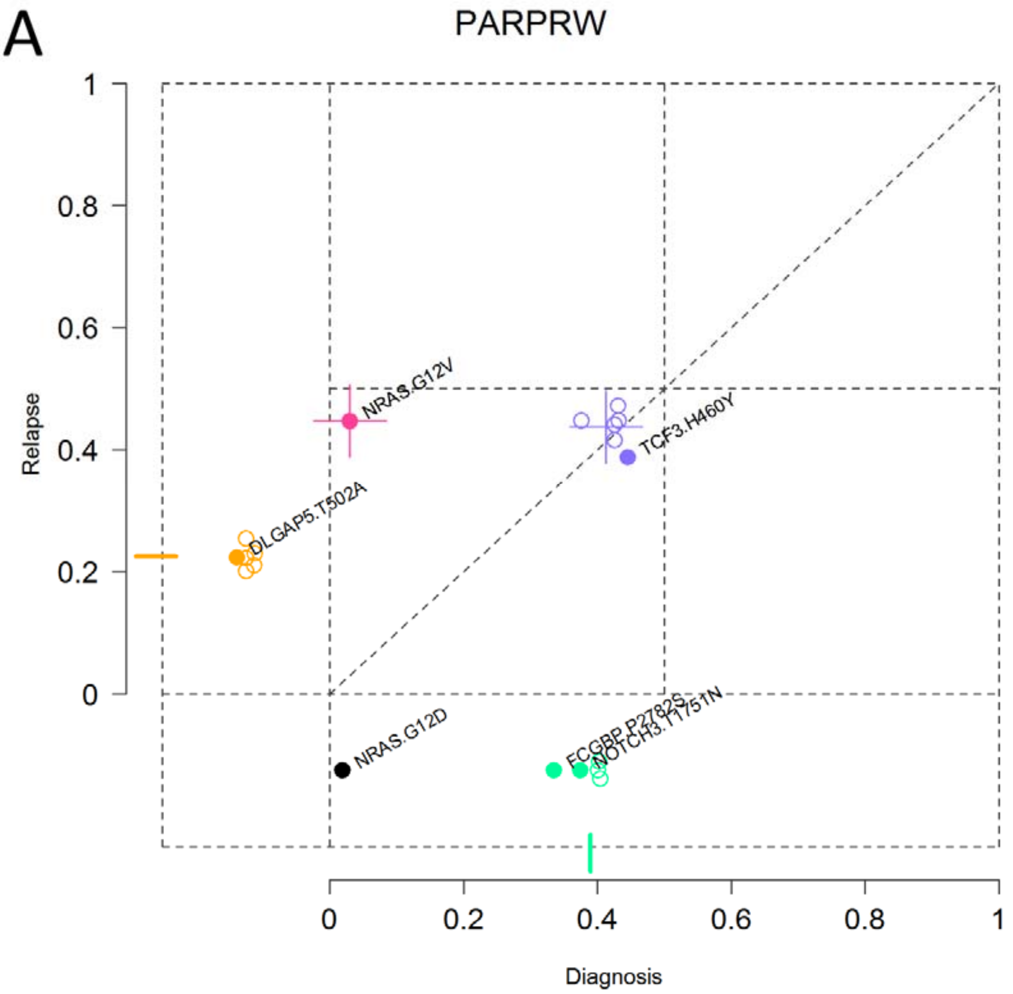
PARPRW
Relapse
Diagnosis
1
0.8
0.6
0.4
0.2
0
0.2
0.4
0.6
0.8
1
NRAS.G12V
TCF3.H460Y
DLGAP5.T502A
NRAS.G12D
FCGBP.P2782S
NOTCH3.T1751N

B

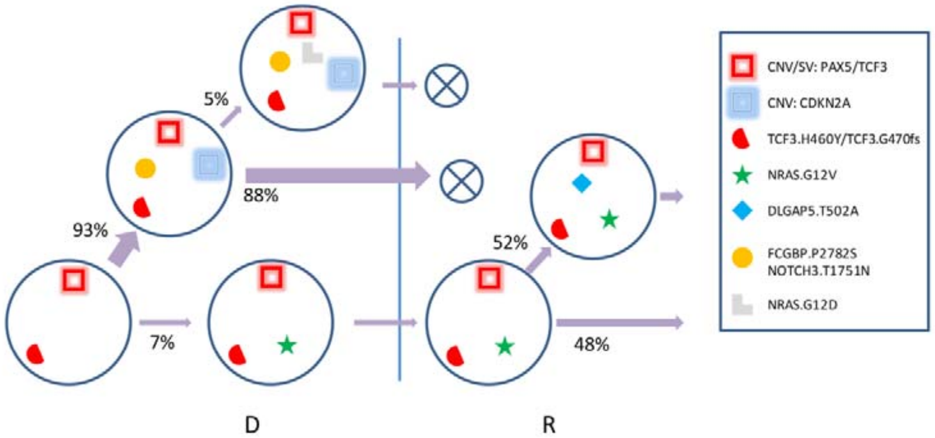
5%
88%
93%
7%
52%
48%
D
R
CNV/SV: PAX5/TCF3
CNV: CDKN2A
TCF3.H460Y/TCF3.G470fs
NRAS.G12V
DLGAP5.T502A
FCGBP.P2782S
NOTCH3.T1751N
NRAS.G12D

Supplementary Figure 9. Clonal architecture of diagnostic (D) and relapsed (R) tumors for PARPRW.

A) Scatter plot of mutant allele fraction (MAF) of diploid SNVs. B) At diagnosis, *TCF3-HLF,* deletion of *PAX5*, and a *TCF3* p.His460Tyr mutation were present in the founder clone. The predominant clone that accounted for over 90% tumor cells at diagnosis acquired additional lesions including *CDKN2A* deletion and mutations *FCGBP* p.Pro2782Ser and *NOTCH3* p.Thr1751Asn. The predominant clone and its descendant subclone with *NRAS* p.Gly12Asp were eradicated by therapy. A minor subclone which accounted for 7% of the tumor cells from diagnosis harbors a *NRAS* p.Gly12Val mutation. It survived therapy and persisted to relapse. The relapsed tumor consists of this clone along with its descendant subclone that acquired additional mutations represented by a p.Thr520Ala mutation in *DLGAP5*.

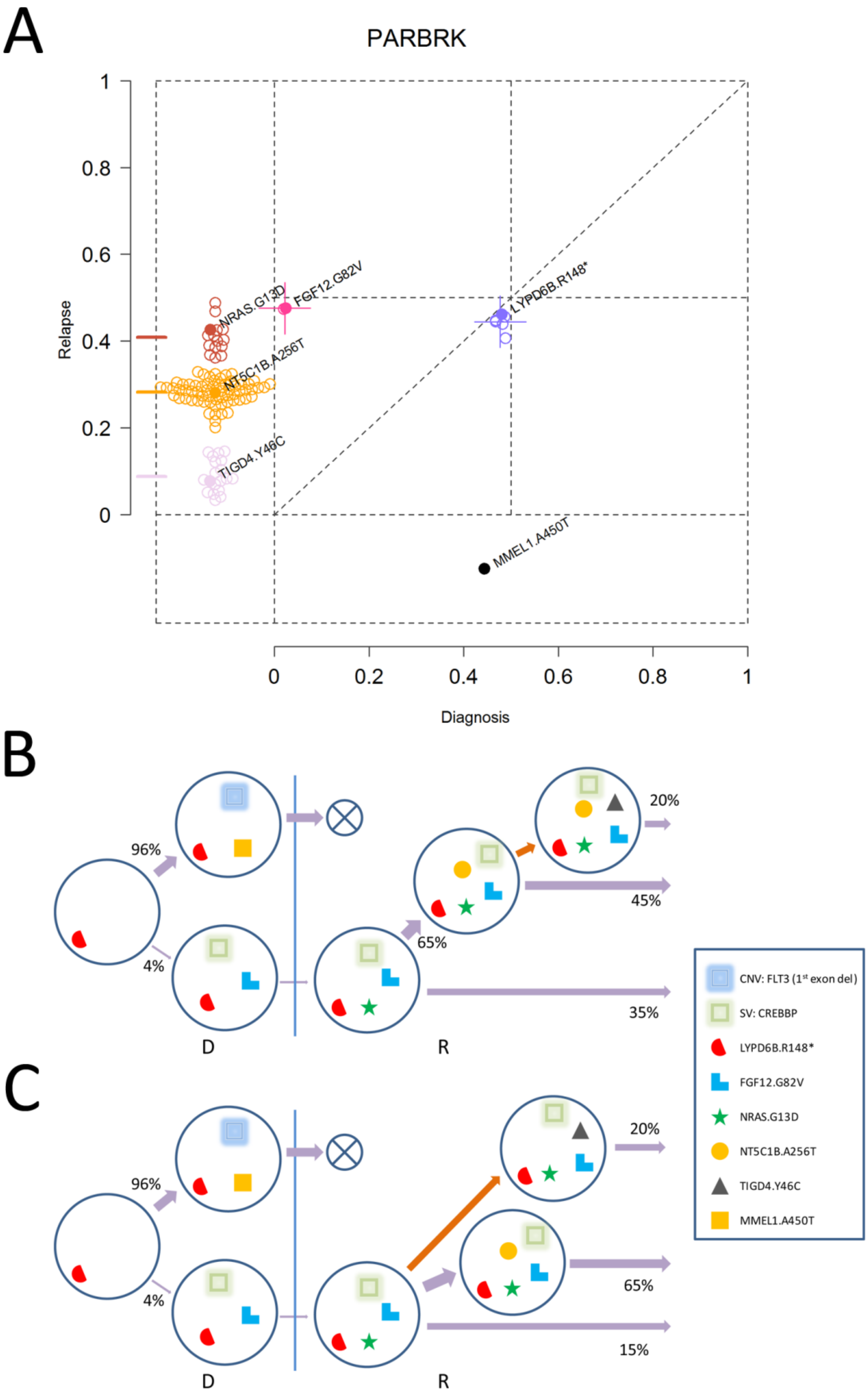

A
PARBRK
Relapse
Diagnosis
NRAS.G13D
FGF12.G82V
NT5C1B.A256T
TIGD4.Y46C
LYPD6B.R148*
MMEL1.A450T
B
96%
4%
65%
20%
45%
35%
D
R
C
96%
4%
20%
65%
15%
D
R
CNV: FLT3 (1st exon del)
SV: CREBBP
LYPD6B.R148*
FGF12.G82V
NRAS.G13D
NT5C1B.A256T
TIGD4.Y46C
MMEL1.A450T

Supplementary Figure 10. Clonal architecture of diagnostic (D) and relapsed (R) tumors for PARBRK.

A) Scatter plot of mutant allele fraction (MAF) of diploid SNVs. B) At diagnosis, a mutation cluster labeled by *LYPD6B* p.Arg148Ter is present in the ancestral founder clone. The predominant clone that account for >95% tumor cells at diagnosis is a descendant of the ancestral clone and contains additional lesions including a focal deletion in *FLT3* and a *MMEL1* p.Ala450Thr mutation. This predominant clone was eradicated by therapy. A minor subclone which accounted for 4% of the tumor cells at diagnosis harbors a focal deletion of *CREBBP* which removes amino acid 822-1682 of *CREBBP* along with other mutations. This subclone survived therapy and became the relapse founder clone after acquiring an additional *NRAS* p.Gly13Asp mutation at relapse. At relapse, the founder clone has two descendant subclones. The orange arrow in relapse indicates an alternative clonal lineage for *TIGD4* p.Try46Cys as shown in C). Scenario B) is used in Figure 6.

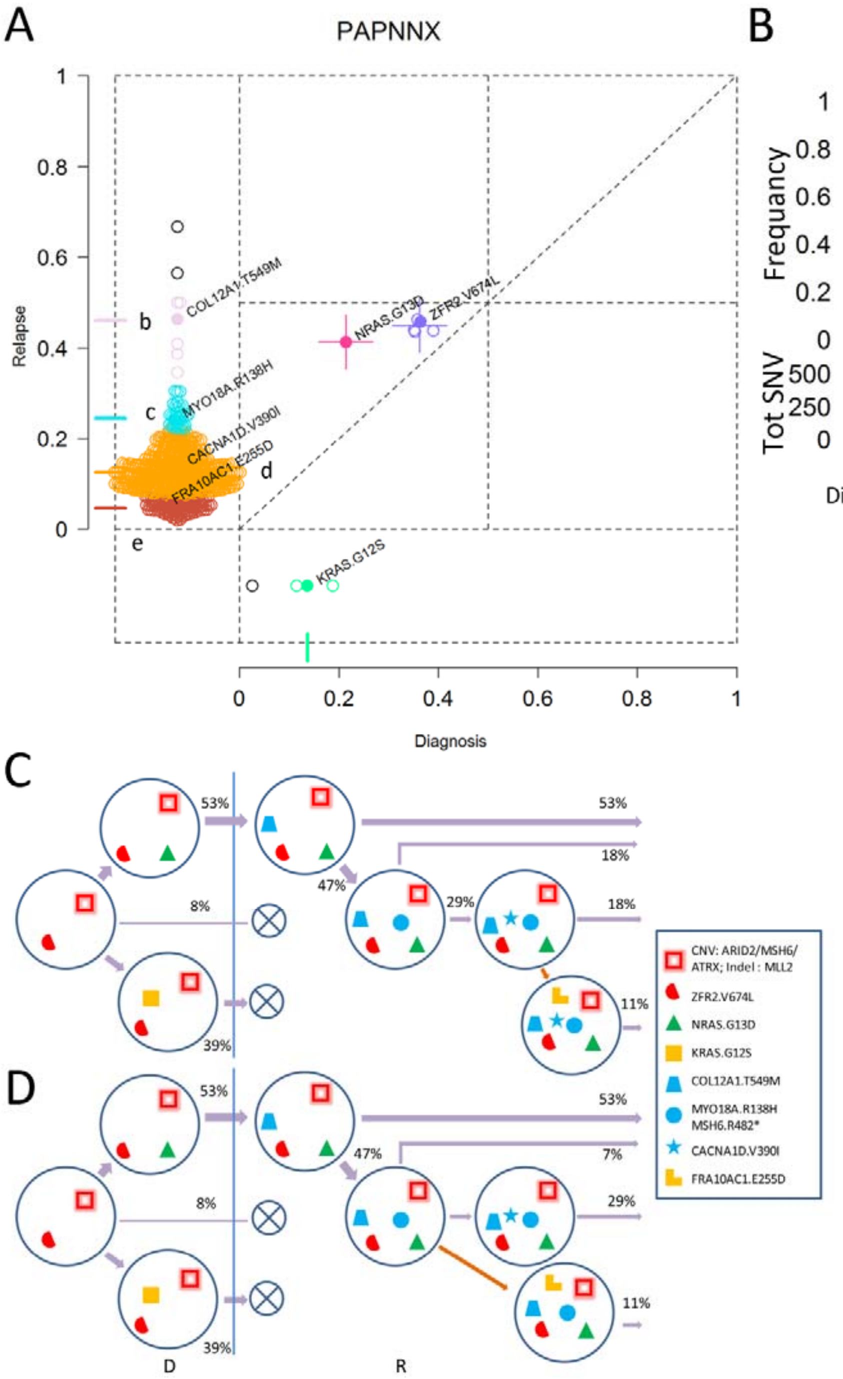
A
PAPNNX
Relapse
Diagnosis
COL12A1.T549M
MYO18A.R138H
CACNA1D.V390I
FRA10AC1.E255D
NRAS.G13D
ZFR2.V674L
KRAS.G12S
B
Frequancy
Tot SNV
Diagnosis
C(G)>T(A)
A(T)>G(C)
C(G)>G(C)
C(G)>A(T)
A(T)>T(A)
A(T)>C(G)
C
D
CNV: ARID2/MSH6/ ATRX; Indel : MLL2
ZFR2.V674L
NRAS.G13D
KRAS.G12S
COL12A1.T549M
MYO18A.R138H MSH6.R482*
CACNA1D.V390I
FRA10AC1.E255D
D
R

Supplementary Figure 11. Clonal architecture of diagnostic (D) and relapsed (R) tumors for PAPNNX.

A) Scatter plot of mutant allele fraction (MAF) of diploid SNVs. B) The mutation spectrum in relapse subclones (indicated by **b**, **c**, **d**, **e**) showed an enrichment of transition mutations as compared to that in diagnosis subclones. C) At diagnosis, heterozygous deletions of *ARID2*, *MSH6* and ATRX and an *MLL2* indel mutation were present in the founder clone. This founder clone had two descendant clones. One was a minor clone which acquired an additional *KRAS* p.Gly12Ser mutation. It accounted for 39% of the tumor cells at diagnosis and was eliminated by therapy. The other was a predominant clone which acquired a *NRAS* p.Gly13Asp mutation. This predominant clone accounted for 53% of the tumor cells at diagnosis and persisted to become the founder clone at relapse. At relapse, its descendant subclone acquired a *MSH6* p.Arg482Ter (labeled by *MYO18A* p.Arg138His) mutation resulting in hypermutation of subclones (**c**, **d**, and **e**) descended from this lineage. The relapse subclone *FRA10AC1* (indicated by orange arrow) had an alternative lineage as shown in D). The subclone *FRA10AC1* would also be assigned as a subclone of relapse founder if its mutation spectrum was not enriched with transitions. Scenario C) is used in Figure 6.

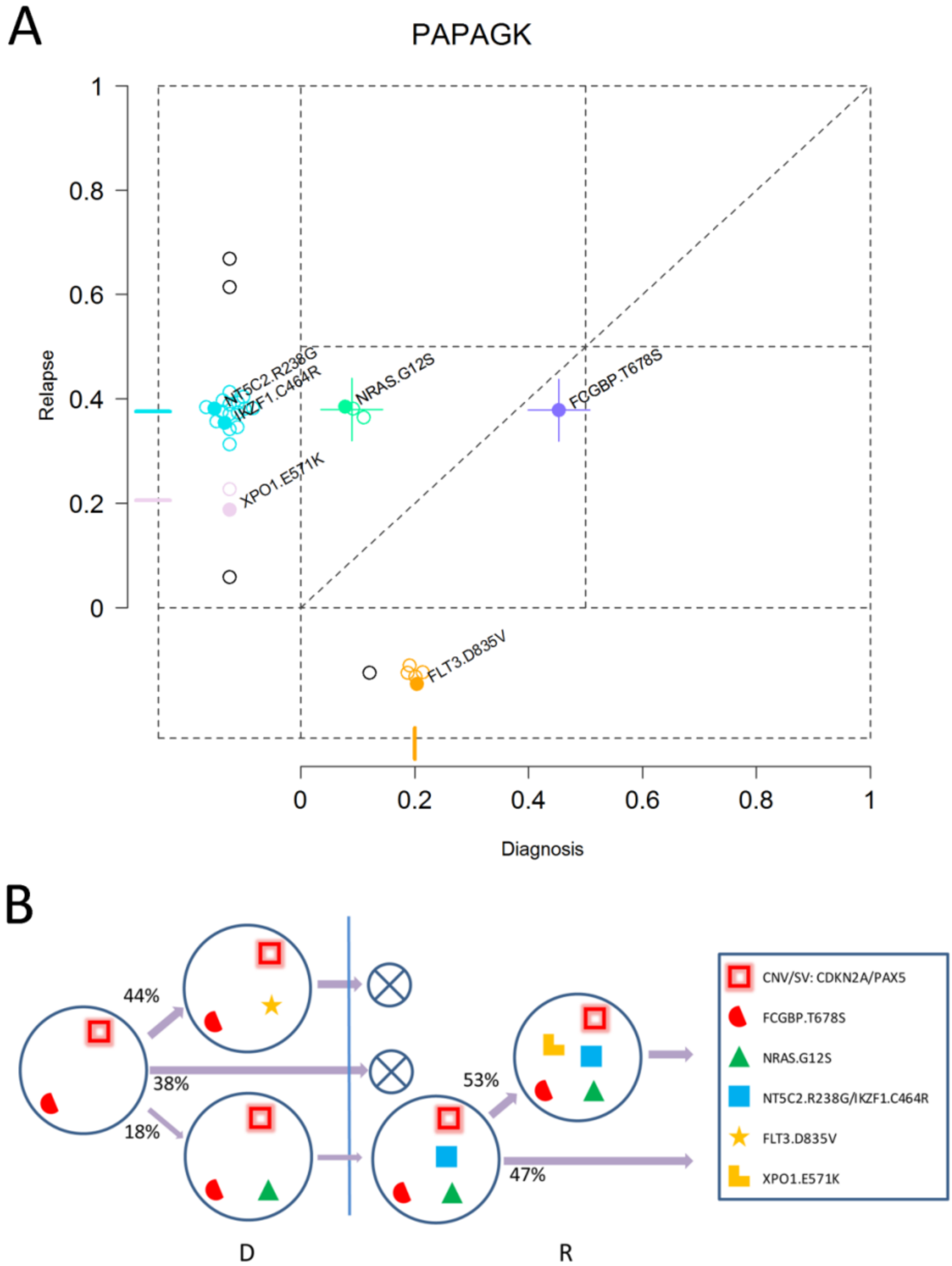
A
PAPAGK
Relapse
Diagnosis
1
0.8
0.6
0.4
0.2
0
NT5C2.R238G
IKZF1.C464R
NRAS.G12S
FCGBP.T678S
XPO1.E571K
FLT3.D835V
0
0.2
0.4
0.6
0.8
1
B
44%
38%
18%
53%
47%
D
R
CNV/SV: CDKN2A/PAX5
FCGBP.T678S
NRAS.G12S
NT5C2.R238G/IKZF1.C464R
FLT3.D835V
XPO1.E571K

Supplementary Figure 12. Clonal architecture of diagnostic (D) and relapsed (R) tumors for PAPAGK.

A) Scatter plot of mutant allele fraction (MAF) of diploid SNVs. B) At diagnosis, deletions of *CDKN2A* and *PAX5* along with a mutation cluster represented by *FCGBP* p.Thr678Ser mutation were present in the founder clone. This founder clone had two descendant subclones. One acquired an additional *FLT3* p.Asp835Val mutation. It accounted for 44% of the tumor cells at diagnosis and was eradicated by therapy. The other was a minor subclone that acquired a *NRAS* p.Gly12Ser mutation. This subclone accounted for 18% of the tumor cells at diagnosis and persisted to become the founder clone at relapse after acquired mutations in *NT5C2* and *IKZF1*. At relapse, it also has a descendant clone that acquired additional mutations including *XPO1* p.Glu571Lys.

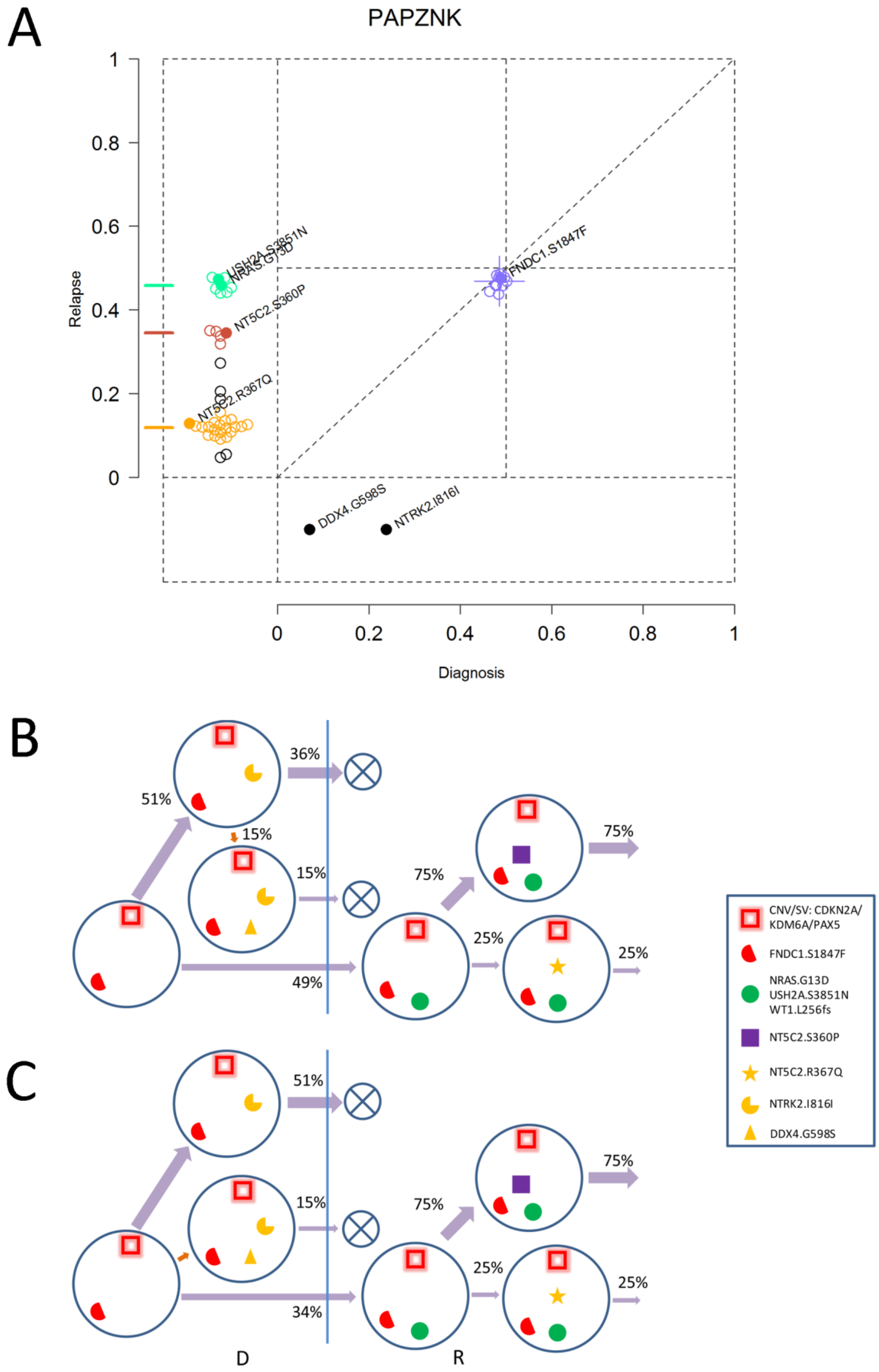
A
PAPZNK
Relapse
Diagnosis
USH2A.S3851N
NRAS.G13D
NT5C2.S360P
NT5C2.R367Q
FNDC1.S1847F
DDX4.G598S
NTRK2.I816I
B
C
D
R
CNV/SV: CDKN2A/ KDM6A/PAX5
FNDC1.S1847F
NRAS.G13D USH2A.S3851N WT1.L256fs
NT5C2.S360P
NT5C2.R367Q
NTRK2.I816I
DDX4.G598S

Supplementary Figure 13. Clonal architecture of diagnostic (D) and relapsed (R) tumors for PAPZNK.

A) Scatter plot of mutant allele fraction (MAF) of diploid SNVs. B) At diagnosis, deletions of *CDKN2A*, *KDM6A* and *PAX5* together with a mutation cluster represented by *FNDC1* p.Ser1847Phe mutation were present in the ancestral clone. Two subclones (*NTRK2* p.Ile816Ile and *DDX4* p.Gly598Ser) coexisted with the ancestral clone at diagnosis. Subclone *DDX4* p.Gly598Ser accounted for 15% of the diagnosis tumor cells and has ambiguous clonal lineage (indicated by orange arrow) as its frequency allowed it to be assigned as a descendant of the subclone with the *NTRK2* mutation or that of the ancestral clone as shown in C). The ancestral clone persisted to relapse and became the relapse founder clone after acquiring mutations in *NRAS*, *USH2A* and *WT1*. The relapse founder clone had two additional subclones each with its own distinct *NT5C2* mutation. One subclone acquired a *NT5C2* p.Arg367Gln and accounted for 25% of the tumor cells. The other acquired a *NT5C2* p.Ser360Pro and accounted for 75% of the tumor cells.

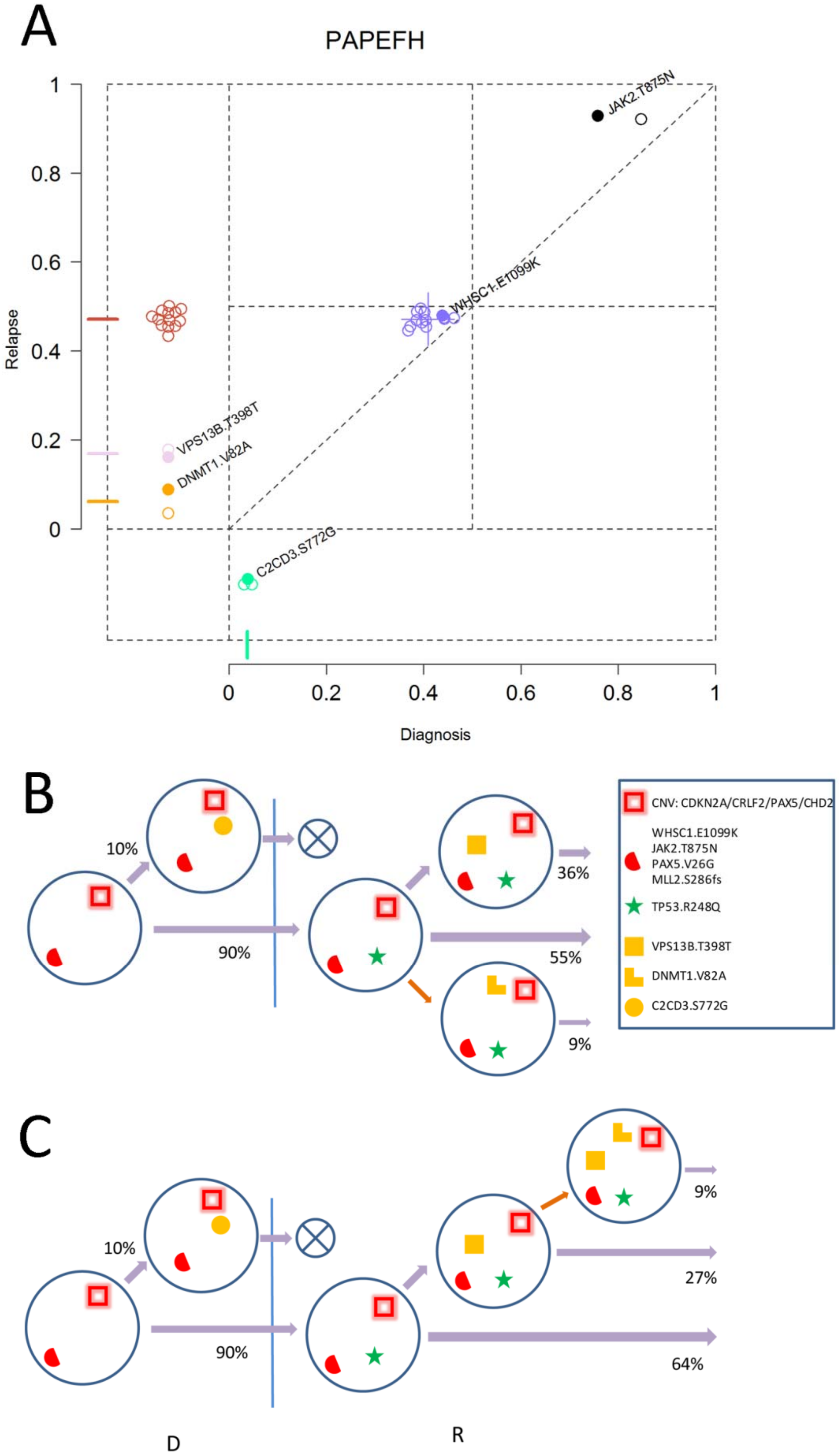
A
PAPEFH
JAK2.T875N
WHSC1.E1099K
VPS13B.T398T
DNMT1.V82A
C2CD3.S772G
Relapse
Diagnosis
0
0.2
0.4
0.6
0.8
1
B
10%
90%
36%
55%
9%
CNV: CDKN2A/CRLF2/PAX5/CHD2
WHSC1.E1099K
JAK2.T875N
PAX5.V26G
MLL2.S286fs
TP53.R248Q
VPS13B.T398T
DNMT1.V82A
C2CD3.S772G
C
10%
90%
9%
27%
64%
D
R

Supplementary Figure 14. Clonal architecture of diagnostic (D) and relapsed (R) tumors for PAPEFH.

A) Scatter plot of mutant allele fraction (MAF) of diploid SNVs. B) At diagnosis, the *P2RY8-CRLF2* fusion, deletions of *CDKN2A*, *PAX5* and *CHD2* along with a mutation cluster including *WHSC1* p.Glu1099Lys, *JAK2* p.Thr875Asn, *PAX5* p.Val26Gly and *MLL2* p.Ser286fs were present in the ancestral founder clone which was also the predominant clone present in 90% of the diagnosis tumor cells. This founder has a descendant subclone that accounted for 10% of the tumor cells with additional mutations. In this case, the predominant clone persisted to relapse and became the founder clone at relapse after acquiring an additional *TP53* p.Arg248Gln mutation. At relapse, this founder clone had two descendant subclones which represent 9% and 36% of the tumor cells at relapse, respectively. Relapse subclone *DNMT1* (indicated by the orange arrow) has an alternative clonal lineage as shown in C). Scenario B) is used in Figure 6.

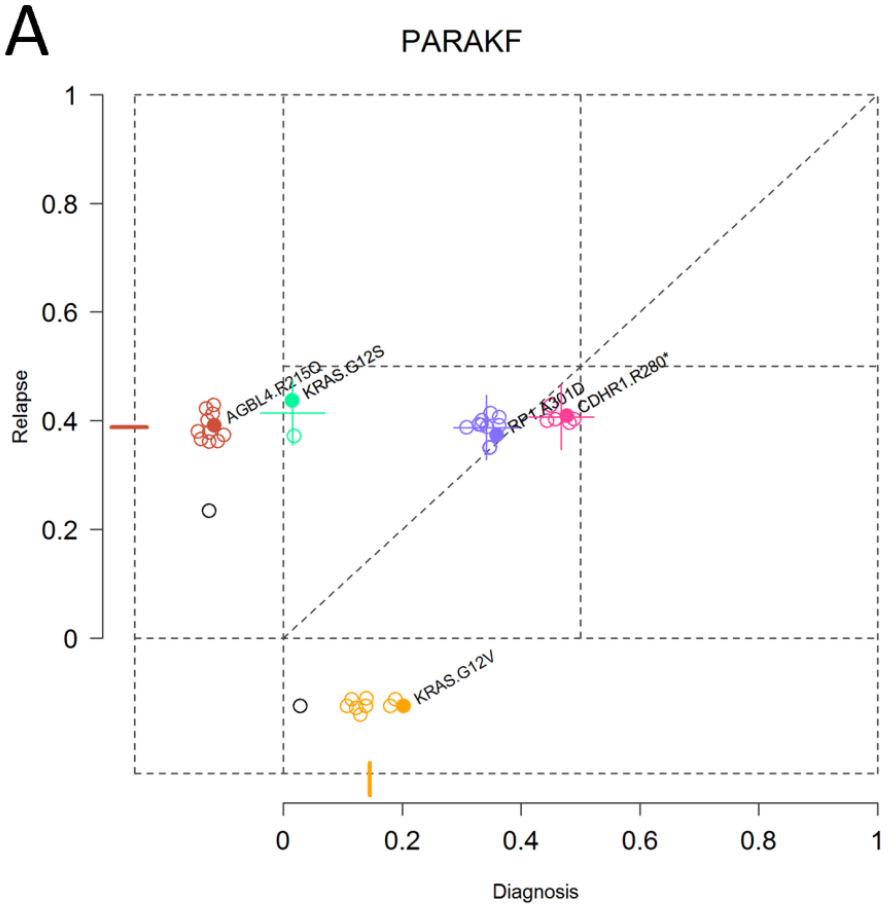
A
PARAKF
Relapse
Diagnosis
AGBL4.R215Q
KRAS.G12S
RP1.A301D
CDHR1.R280*
KRAS.G12V
0
0.2
0.4
0.6
0.8
1

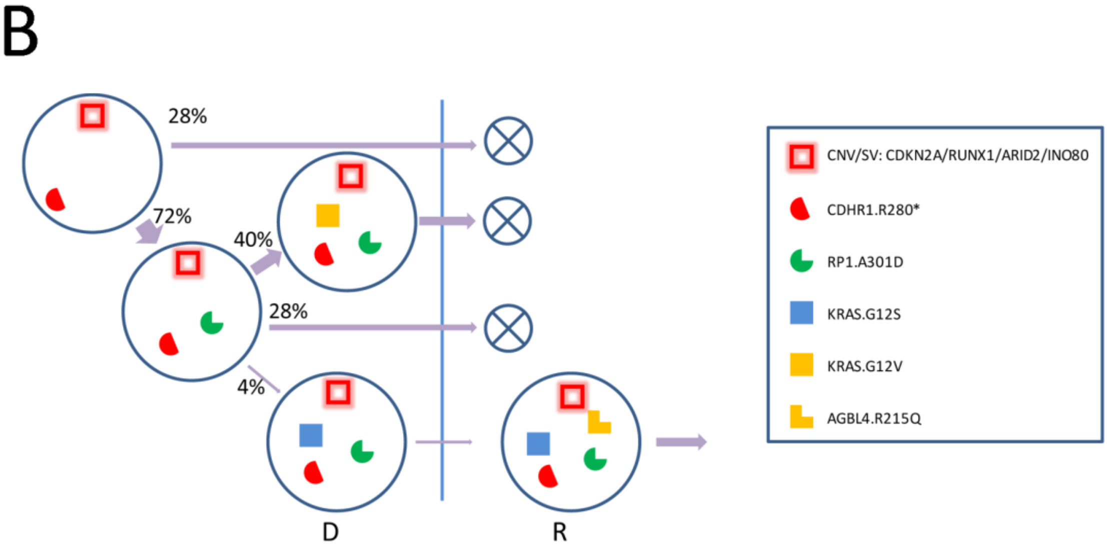
B
28%
72%
40%
28%
4%
D
R
CNV/SV: CDKN2A/RUNX1/ARID2/INO80
CDHR1.R280*
RP1.A301D
KRAS.G12S
KRAS.G12V
AGBL4.R215Q

Supplementary Figure 15. Clonal architecture of diagnostic (D) and relapsed (R) tumors for PARAKF.

A) Scatter plot of mutant allele fraction (MAF) of diploid SNVs. B) At diagnosis, the *ETV6-RUNX1* fusion and deletions of *CDKN2A*, *ARID2* and *INO80* were present in the ancestral founder clone. This founder has three descendant subclones, two of which harbor *KRAS* mutations. The subclone that had *KRAS* p.Gly12Val mutation accounted for 40% of the tumor cells at diagnosis and it was eliminated by therapy. By contrast, the subclone that had *KRAS* p.Gly12Ser mutation accounted for 4% of the tumor cells at diagnosis. It became the founder clone at relapse after acquiring additional relapse-specific mutations labeled by *AGBL4* mutation.

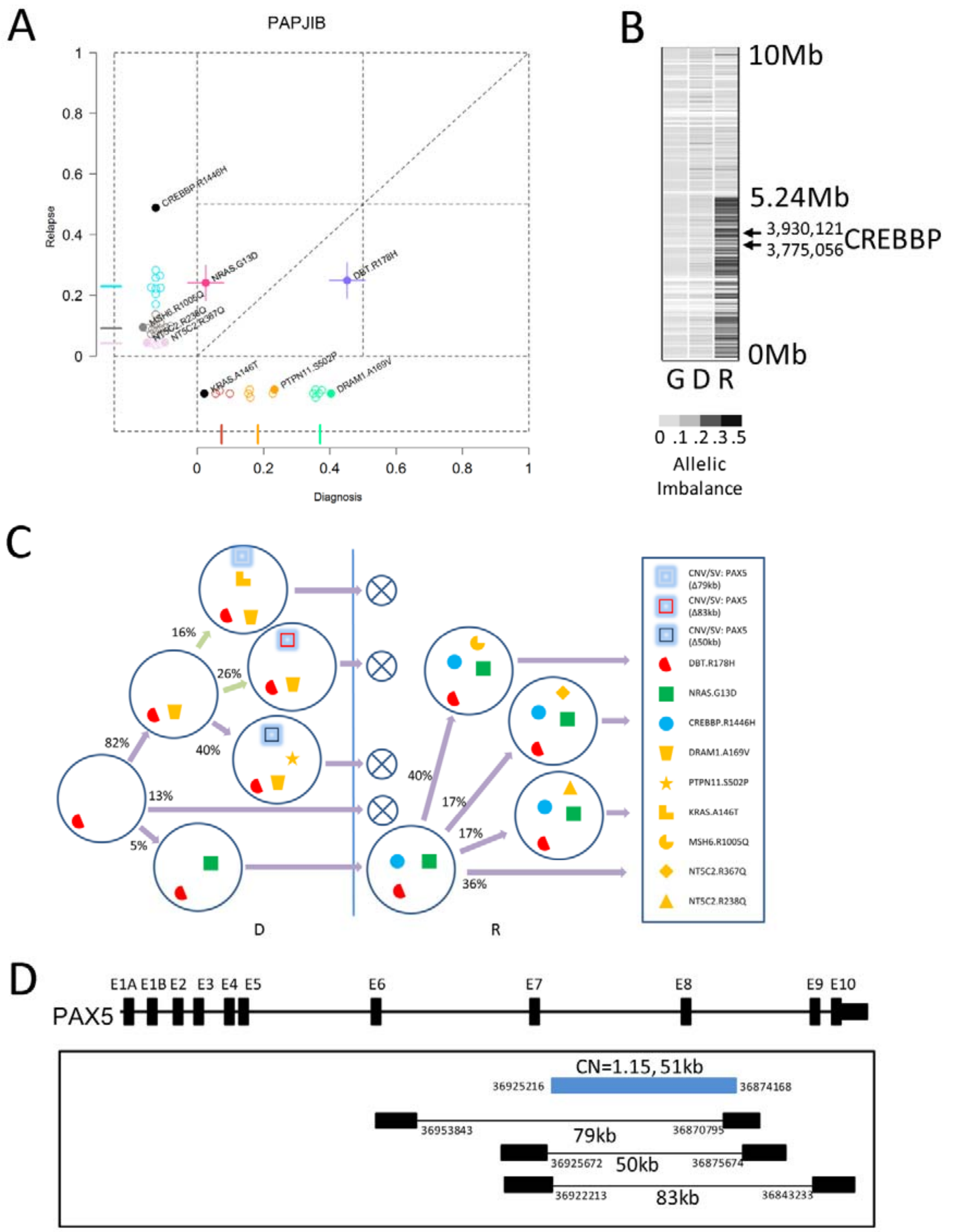

A
PAPJIB
Relapse
Diagnosis
CREBBP.R1446H
NRAS.G13D
DBT.R178H
MSH6.R1005Q
NT5C2.R238Q
NT5C2.R367Q
KRAS.A146T
PTPN11.S502P
DRAM1.A169V
B
10Mb
5.24Mb
3,930,121
3,775,056
CREBBP
0Mb
G D R
0 .1 .2 .3 .5
Allelic Imbalance
C
CNV/SV: PAX5 (Δ79kb)
CNV/SV: PAX5 (Δ83kb)
CNV/SV: PAX5 (Δ50kb)
DBT.R178H
NRAS.G13D
CREBBP.R1446H
DRAM1.A169V
PTPN11.S502P
KRAS.A146T
MSH6.R1005Q
NT5C2.R367Q
NT5C2.R238Q
82%
16%
26%
40%
13%
5%
40%
17%
17%
36%
D
R
D
PAX5
E1A E1B E2 E3 E4 E5 E6 E7 E8 E9 E10
CN=1.15, 51kb
36925216
36874168
36953843
79kb
36870795
36925672
50kb
36875674
36922213
83kb
36843233

Supplementary Figure 16. Clonal architecture of diagnostic (D) and relapsed (R) tumors for PAPJIB.

A) Scatter plot of mutant allele fraction (MAF) of diploid SNVs. B) Allelic imbalance of chr16:1-5.24Mb encompassing the CREBBP locus in the relapsed tumor. The allelic imbalance is shown using the same gray scale as Supplementary Fig. 6 which indicates copy-neutral LOH in this region. Only germline SNVs from CGI WGS data with coverage >20x in G and >20x in D and R samples are included. Relapsed tumor has 45% tumor purity (Supplementary Data 1), therefore the 0.49 MAF value of mutation *CREBBP* p.Arg1446His in panel A indicates bi-allelic loss of CREBBP in tumor. C) At diagnosis, three distinct subclonal SVs in *PAX5* (panel D) were identified along with 3 subclonal mutations in the Ras signaling pathway: *NRAS* p.Gly13Asp, *PTPN11* p.Ser502Pro and *KRAS* p.Ala146Thr. All but the subclone that had the *NRAS* p.Gly13Asp mutation, which did not have a *PAX5* SV, persisted to relapse. This subclone accounted for approximately 5% of the tumor cells at diagnosis. It became the founder clone at relapse after acquiring relapse-specific mutation *CREBBP* p.Arg1446His.The relapse founder clone has 3 descendant subclones including two distinct *NT5C2* mutations, p.Arg367Gln and p.Arg238Gln. We assigned the two *NT5C2* mutations as daughter clones of relapse founder based on mutual exclusivity rule. The assignment of the two *PAX5* SVs (Δ79kb and Δ83kb; indicated by green arrows) in diagnosis sample is ambiguous. D) Focal deletions of *PAX5* in PAPJIB diagnosis sample. Gene model is illustrated in top panel with exons depicted by thick boxes, indexed by exon 1A through exon 10. Copy number changes detected by SNP6 array was indicated by a blue box. Structural variations were indicated by a thin line connecting two filled black boxes. Genomic coordinates of the events are also indicated.

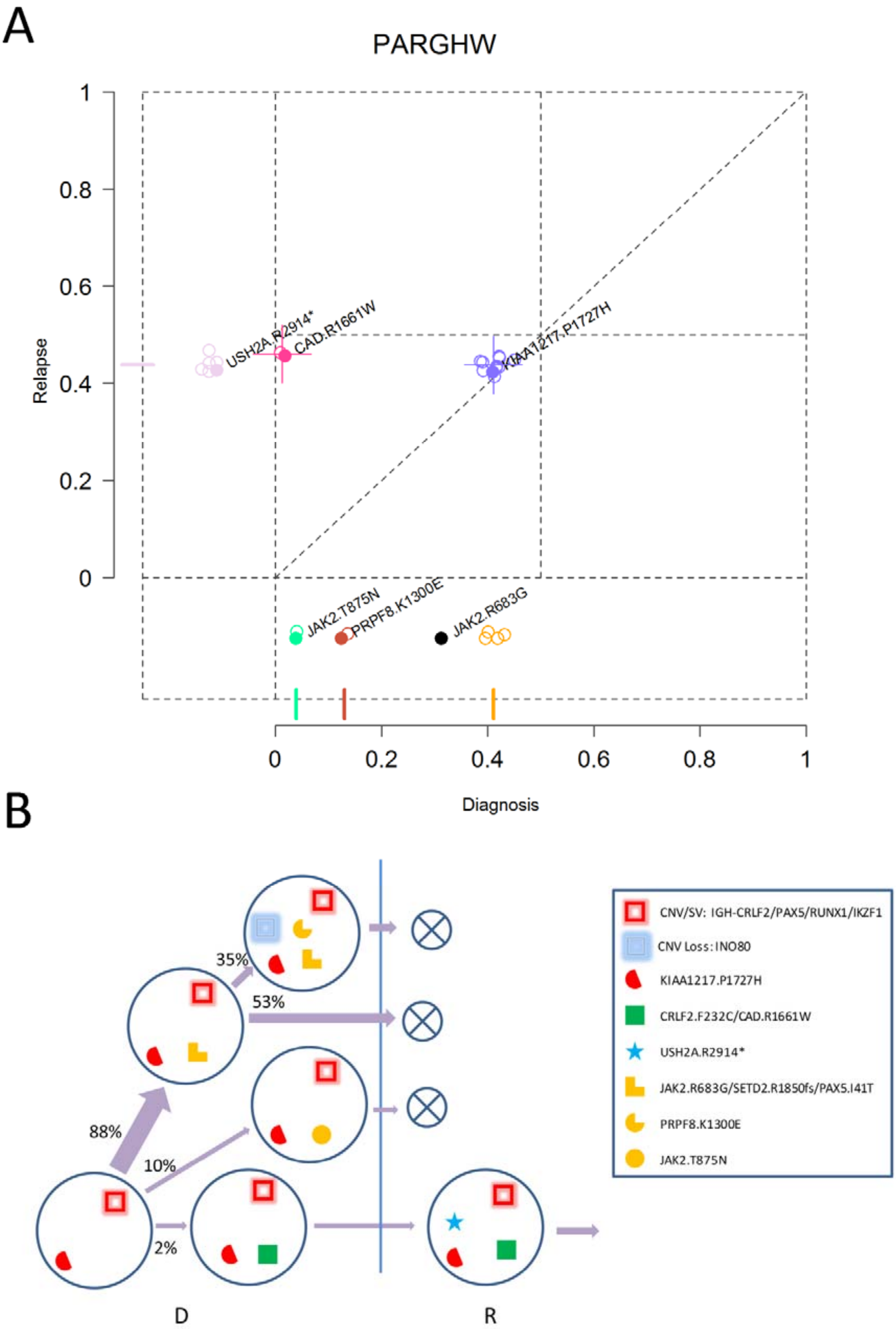
A
PARGHW
Relapse
Diagnosis
1
0.8
0.6
0.4
0.2
0
USH2A.R2914*
CAD.R1661W
KIAA1217.P1727H
JAK2.T875N
PRPF8.K1300E
JAK2.R683G
B
35%
53%
88%
10%
2%
D
R
CNV/SV: IGH-CRLF2/PAX5/RUNX1/IKZF1
CNV Loss: INO80
KIAA1217.P1727H
CRLF2.F232C/CAD.R1661W
USH2A.R2914*
JAK2.R683G/SETD2.R1850fs/PAX5.I41T
PRPF8.K1300E
JAK2.T875N

Supplementary Figure 17. Clonal architecture of diagnostic (D) and relapsed (R) tumors for PARGHW.

A) Scatter plot of mutant allele fraction (MAF) of diploid SNVs. B) At diagnosis, *IGH-CRLF2* rearrangement together with deletions of *PAX5*, *RUNX1* and *IKZF1* were present in the founder clone. Of its 4 descendant subclones, 3 had activating *JAK2* mutations (p.Arg683Gly or p.Thr875Asn), none of which survived therapy. However, the only subclone that persisted to relapse had a *CRLF2* p.Phe232Cys mutation (labeled by *CAD* p.Arg1661Trp) at diagnosis. This subclone accounted for 2% of the tumor cells at diagnosis and became the founder and the dominant clone at relapse after acquired a relapse-specific *USH2A* p.Arg2914X mutation. In diagnosis, both of *JAK2* subclones were assigned as a daughter clone of founder *KIAA1217* based on mutual exclusivity rule.

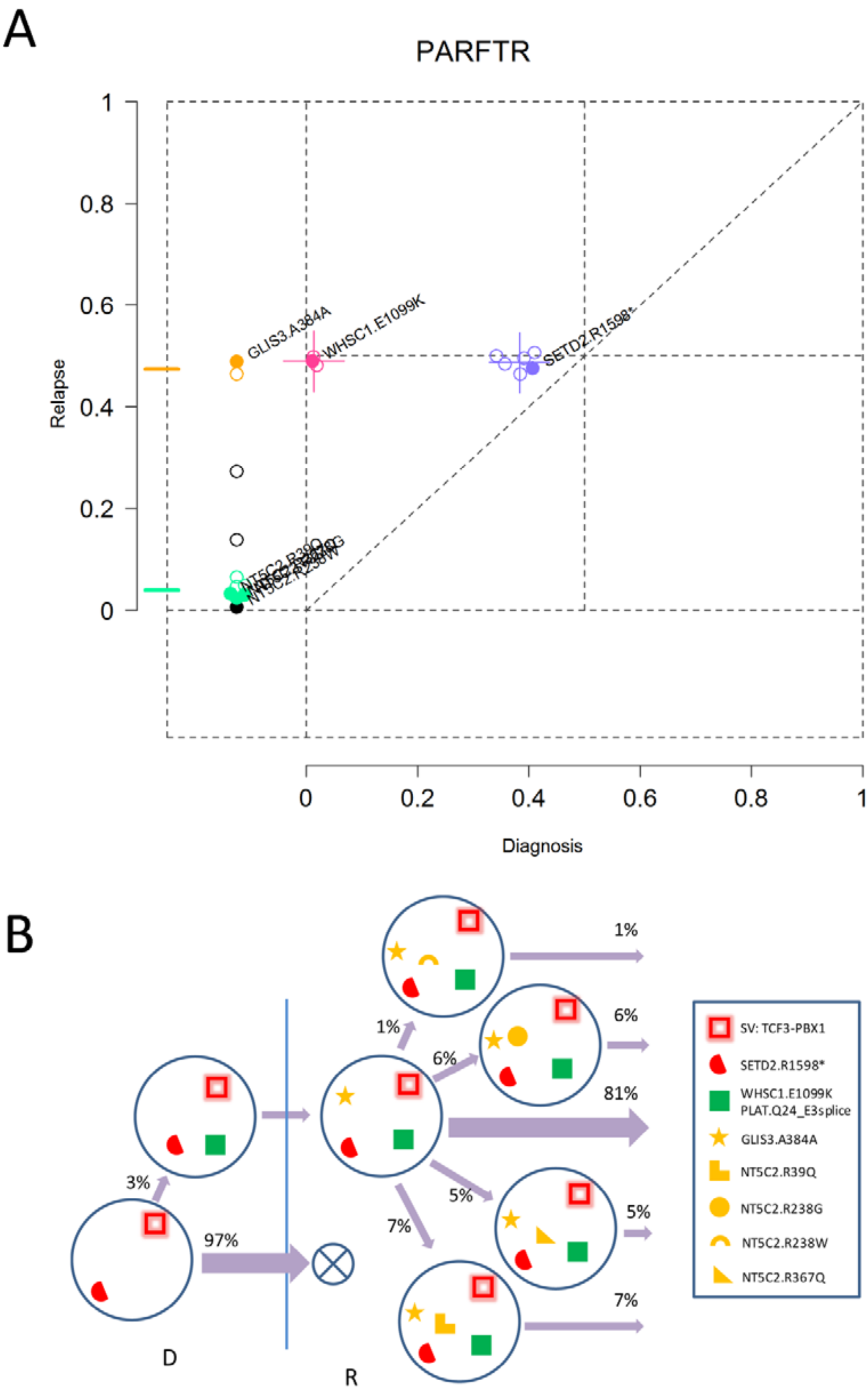
A
PARFTR
Relapse
Diagnosis
1
0.8
0.6
0.4
0.2
0
0
0.2
0.4
0.6
0.8
1
GLIS3.A384A
WHSC1.E1099K
SETD2.R1598*
B
D
R
3%
97%
1%
6%
81%
5%
7%
1%
6%
5%
7%
SV: TCF3-PBX1
SETD2.R1598*
WHSC1.E1099K
PLAT.Q24_E3splice
GLIS3.A384A
NT5C2.R39Q
NT5C2.R238G
NT5C2.R238W
NT5C2.R367Q

Supplementary Figure 18. Clonal architecture of diagnostic (D) and relapsed (R) tumors for PARFTR.

A) Scatter plot of mutant allele fraction (MAF) of diploid SNVs. B) At diagnosis, the *TCF3-PBX1* fusion along with a mutation cluster represented by *SETD2* p.Arg1598Ter were present in the ancestral founder clone. A descendant minor subclone from the founder clone acquired an additional mutation *WHSC1* p.Gln1099Lys and accounted for 3% of tumor cells at diagnosis. The predominant clone was eliminated by therapy while the minor subclone persisted to relapse and acquired additional mutations to become the founder of the relapse. It had four additional subclones with distinct mutations in *NT5C2* (p.Arg39Gln, p.Arg238Trp, p.Arg238Gln, and p.Arg367Gln), which are assigned as daughter clones of relapse founder clone *GLIS3* p.Ala384Ala based on mutual exclusivity rule.

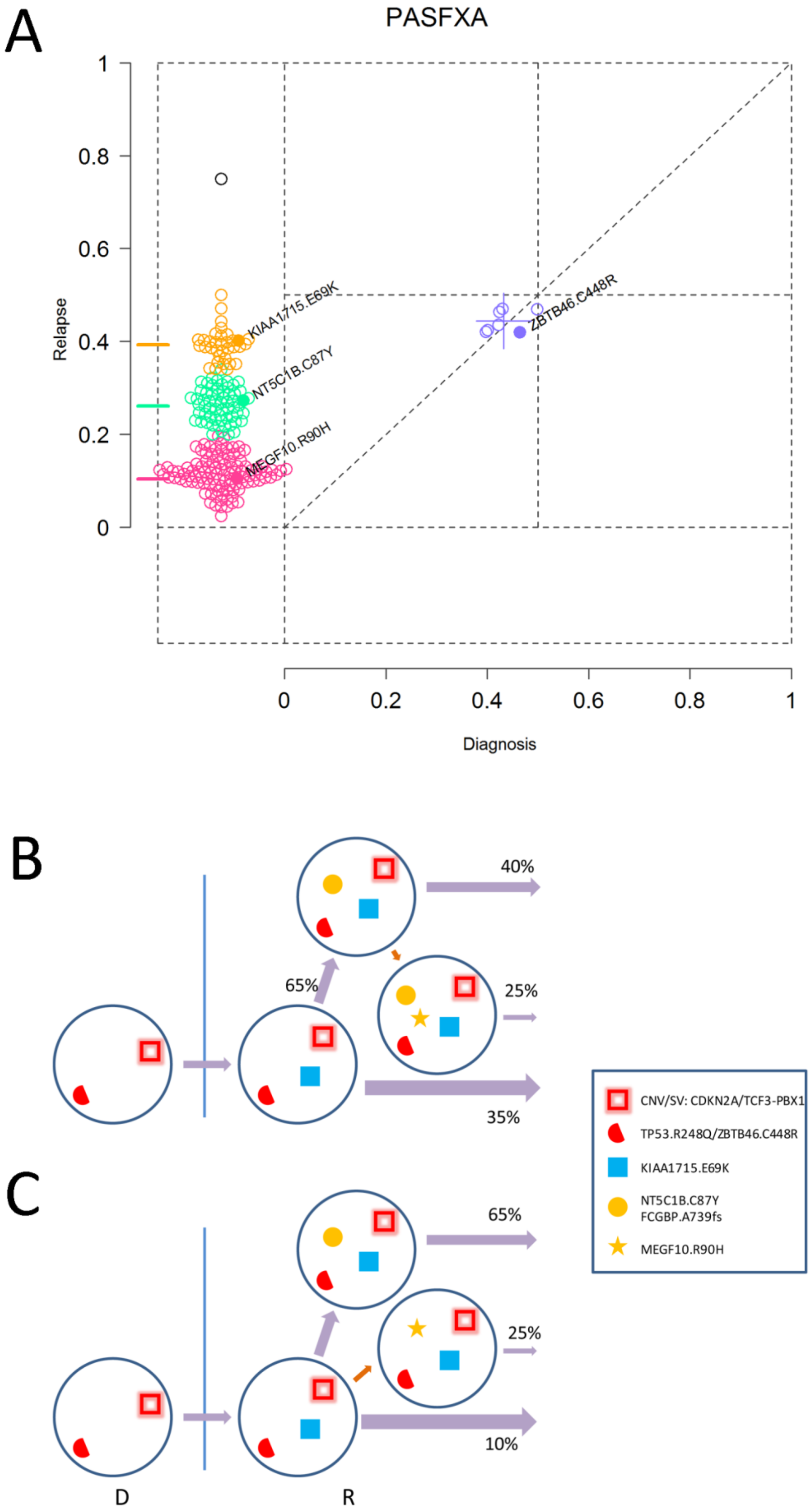
A
PASFXA
1
0.8
0.6
0.4
0.2
0
Relapse
KIAA1715.E69K
NT5C1B.C87Y
MEGF10.R90H
ZBTB46.C448R
0
0.2
0.4
0.6
0.8
1
Diagnosis
B
40%
65%
25%
35%
C
65%
25%
10%
D
R
CNV/SV: CDKN2A/TCF3-PBX1
TP53.R248Q/ZBTB46.C448R
KIAA1715.E69K
NT5C1B.C87Y
FCGBP.A739fs
MEGF10.R90H

Supplementary Figure 19. Clonal architecture of diagnostic (D) and relapsed (R) tumors for PASFXA.

A) Scatter plot of mutant allele fraction (MAF) of diploid SNVs. B) At diagnosis, *TCF3-PBX1* fusion, *CDKN2A* deletion along with a mutation cluster represented by *TP53* p.Arg248Gln mutation were present in the ancestral founder clone. The founder clone persisted to relapse and acquired additional mutations including *FCGBP* p.Ala739fs. The relapse founder had two subclones, labeled by *NT5CB1* p.Cys87Tyr/*FCGBP* p.Ala739fs and *MEGF10* p.Arg90His. Subclone *MEGF10* p.Arg90His (indicated by an orange arrow) had an alternative lineage as shown in C). Scenario B) is used in Figure 6.

A

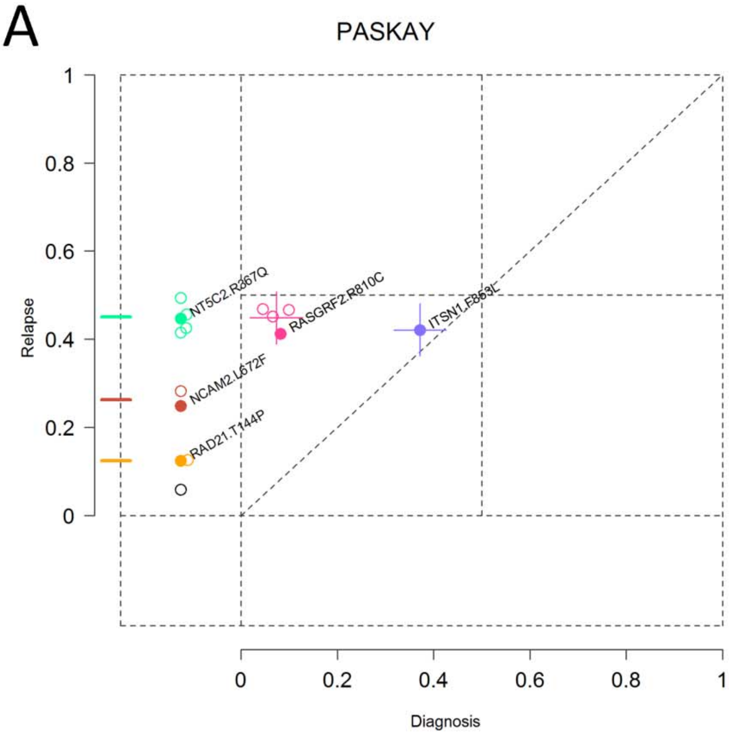
PASKAY
Relapse
Diagnosis
1
0.8
0.6
0.4
0.2
0
0
0.2
0.4
0.6
0.8
1
NT5C2.R367Q
RASGRF2.R810C
ITSN1.F853L
NCAM2.L672F
RAD21.T144P

B

C

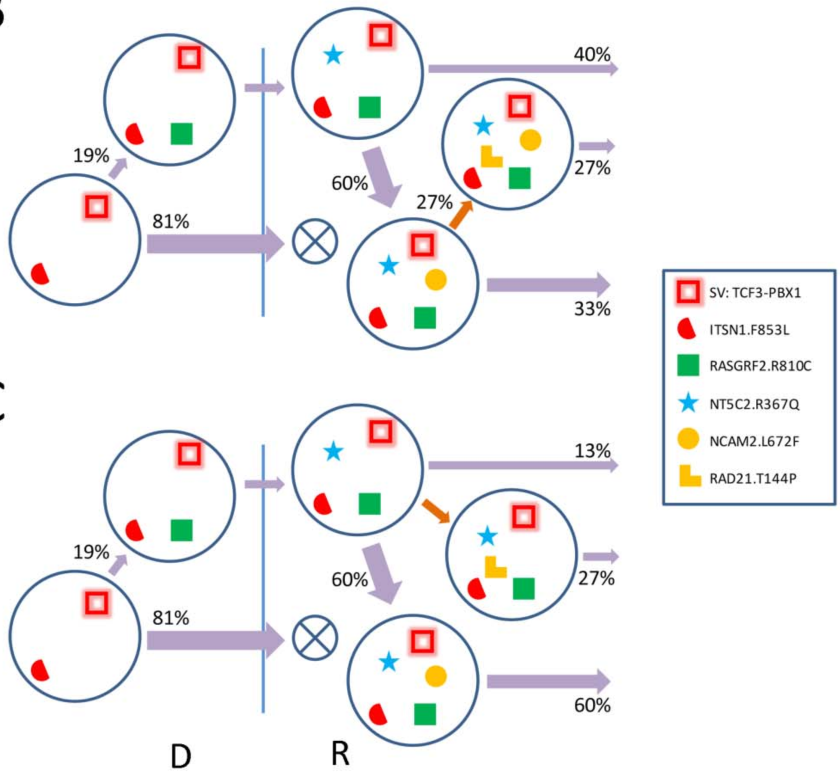
19%
81%
40%
60%
27%
27%
33%
19%
81%
13%
60%
27%
60%
SV: TCF3-PBX1
ITSN1.F853L
RASGRF2.R810C
NT5C2.R367Q
NCAM2.L672F
RAD21.T144P
D
R

Supplementary Figure 20. Clonal architecture of diagnostic (D) and relapsed (R) tumors for PASKAY.

A) Scatter plot of mutant allele fraction (MAF) of diploid SNVs. B) At diagnosis, the *TCF3-PBX1* fusion along with a mutation cluster labeled by *ITSN1* p.Phe853Leu were present in the ancestral founder clone. The founder clone had a minor subclone that acquired the *RASGRF2* p.Arg810Cys mutation which accounted for 19% of the tumor cells at diagnosis. It persisted to relapse after acquired a relapse-specific mutation *NT5C2* p.Arg367Gln. At relapse, it had two additional descendant subclones with distinct mutation clusters. The relapse subclone *RAD21* p.Thr144Pro (indicated with an orange arrow) had an ambiguous lineage. Scenario B) is used in Figure 6.

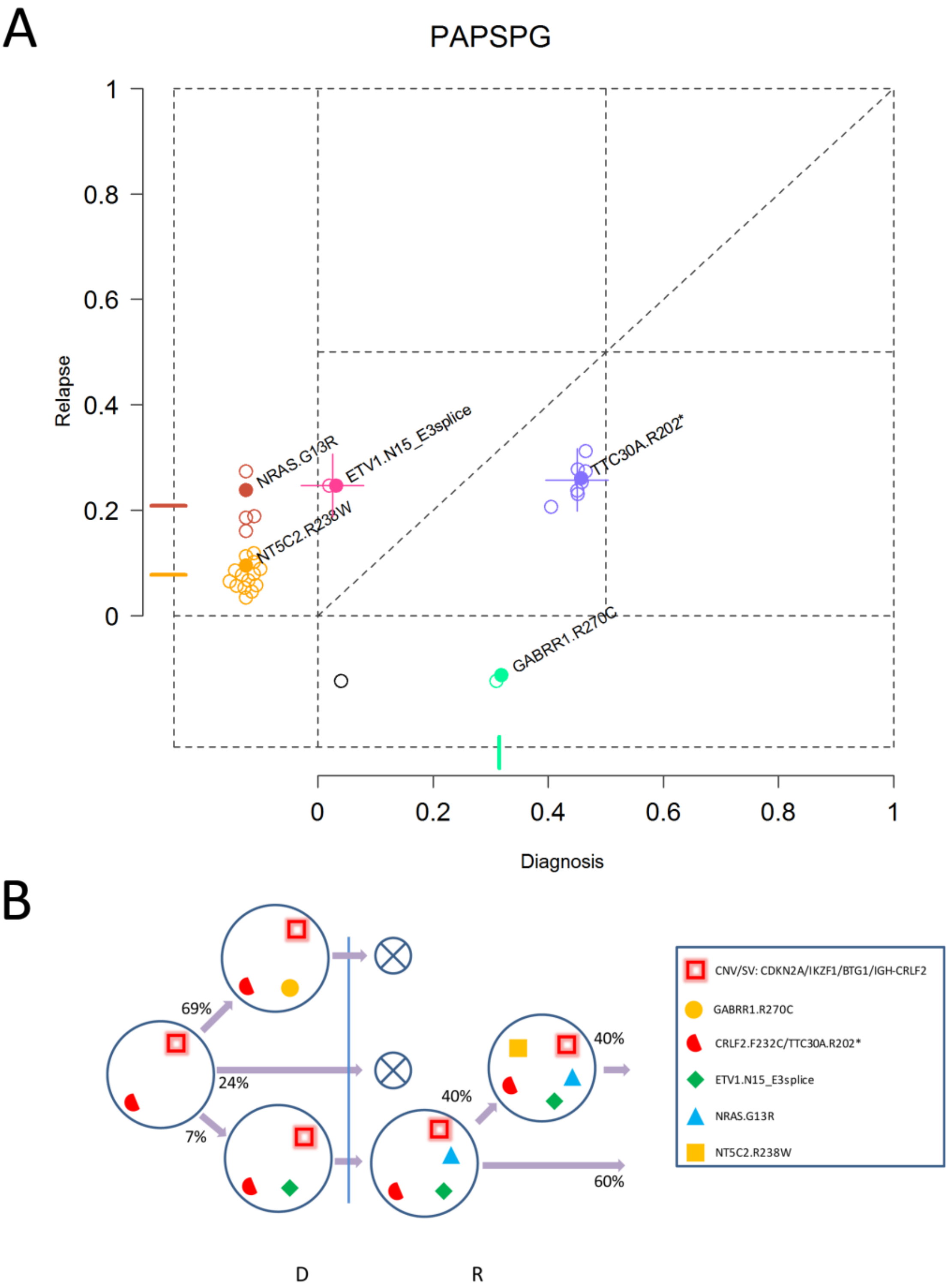
A
PAPSPG
Relapse
Diagnosis
1
0.8
0.6
0.4
0.2
0
0
0.2
0.4
0.6
0.8
1
NRAS.G13R
ETV1.N15_E3splice
NT5C2.R238W
TTC30A.R202*
GABRR1.R270C
B
69%
24%
7%
40%
40%
60%
D
R
CNV/SV: CDKN2A/IKZF1/BTG1/IGH-CRLF2
GABRR1.R270C
CRLF2.F232C/TTC30A.R202*
ETV1.N15_E3splice
NRAS.G13R
NT5C2.R238W

Supplementary Figure 21. Clonal architecture of diagnostic (D) and relapsed (R) tumors for PAPSPG.

A) Scatter plot of mutant allele fraction (MAF) of diploid SNVs. B) At diagnosis, the *IGH-CRLF2* fusion, *CDKN2A* deletion, along with a mutation cluster labeled by *TTC30A* p.Arg202Ter/*CRLF2* p.Phe232Cys were present in the ancestral clone. Of its two daughter clones, the minor subclone *ETV1* p.Asn15_E3splice (accounted for 7% of diagnosis tumor cells) survived the therapy and acquired *NRAS* p.Gly13Arg mutation in relapse, which subsequently gave rise to a subclone *NT5C2* p.Arg238Trp that accounted for 40% of relapse tumor cells.

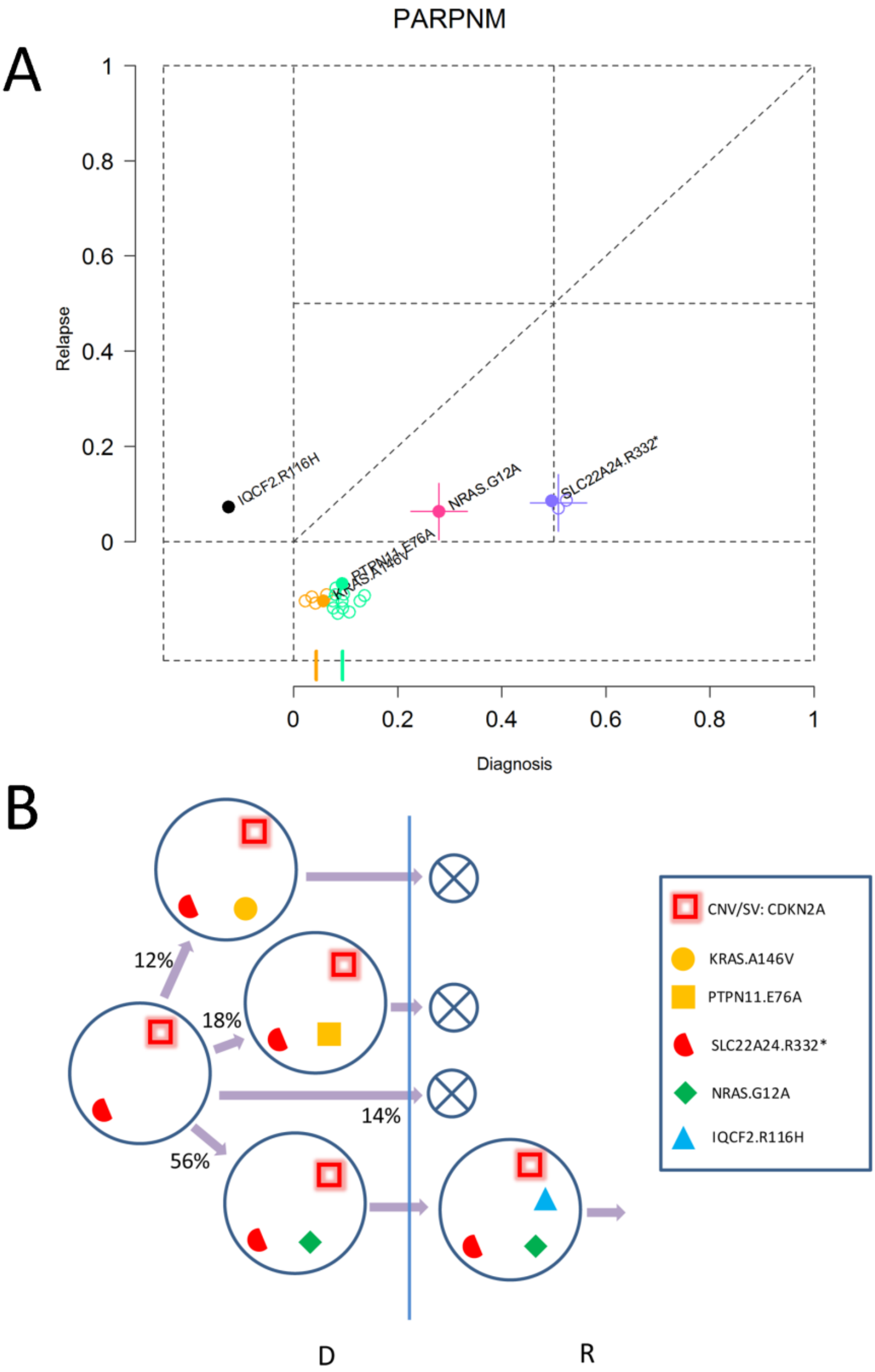
A
PARPNM
Relapse
Diagnosis
0
0.2
0.4
0.6
0.8
1
IQCF2.R116H
NRAS.G12A
SLC22A24.R332*
PTPN11.E76A
KRAS.A146V
B
12%
18%
14%
56%
D
R
CNV/SV: CDKN2A
KRAS.A146V
PTPN11.E76A
SLC22A24.R332*
NRAS.G12A
IQCF2.R116H

Supplementary Figure 22. Clonal architecture of diagnostic (D) and relapsed (R) tumors for PARPNM.

A) Scatter plot of mutant allele fraction (MAF) of diploid SNVs. B) At diagnosis, *CDKN2A* deletion along with a mutation cluster labeled by *SLC22A24* p.Arg332Ter was present in the ancestral founder clone. The founder clone has three subclones, *PTPN11* p.Glu76Ala, *KRAS* p.Ala146Val, and *NRAS* p.Gly12Ala, which were assigned as daughter subclones of the ancestral clone based on mutual exclusivity rule. Only subclone *NRAS* p.Gly12Ala survived the therapy and acquired new mutations including *IQCF2* p.Arg116His in relapse. The low tumor purity (<20%) of relapse sample renders it challenging to detect subclonal lesions in relapse sample and in turn to infer the clonal lineages in relapse sample.

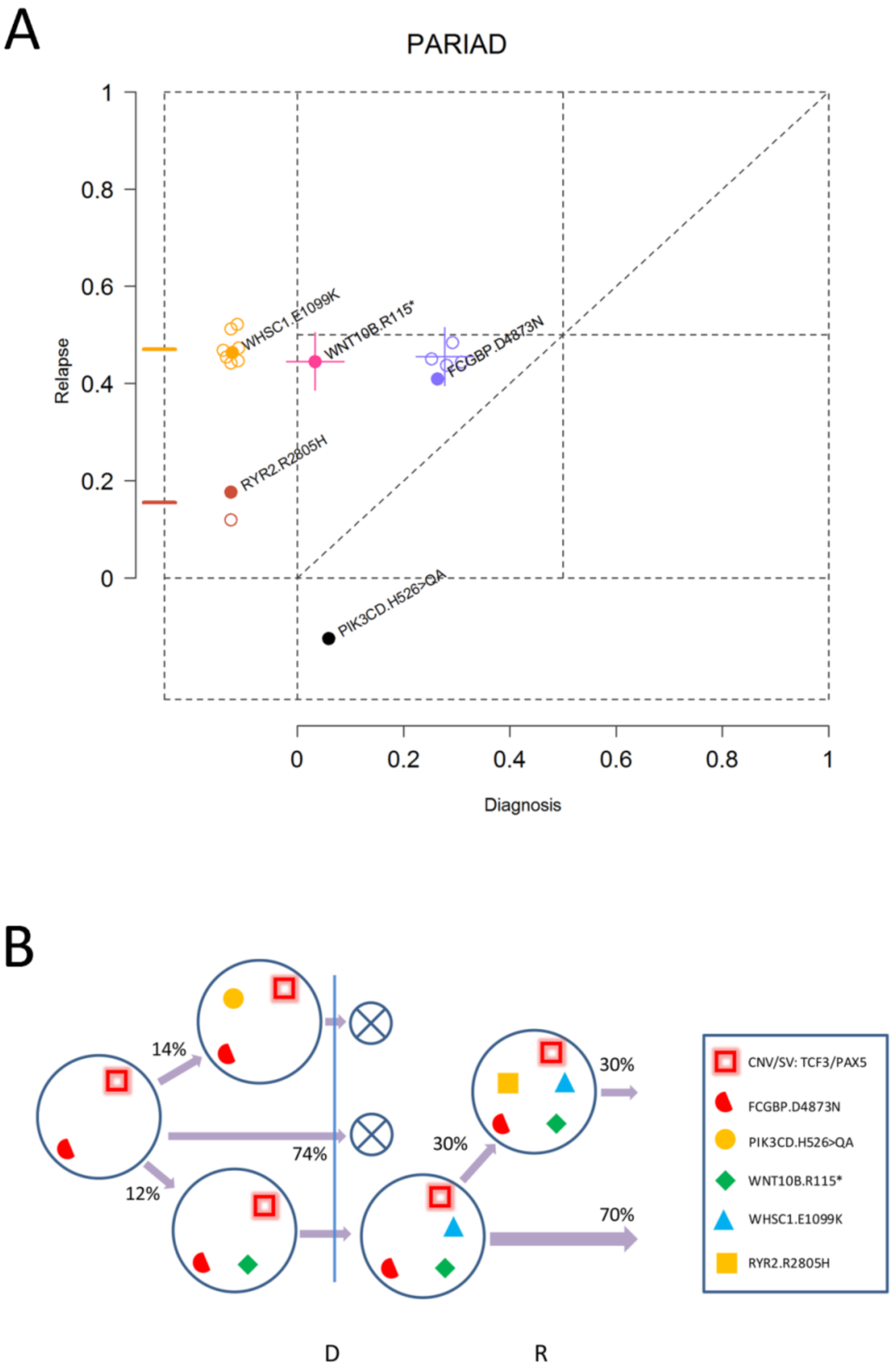
A
PARIAD
Relapse
Diagnosis
0
0.2
0.4
0.6
0.8
1
WHSC1.E1099K
WNT10B.R115*
FCGBP.D4873N
RYR2.R2805H
PIK3CD.H526>QA
B
14%
74%
12%
30%
30%
70%
D
R
CNV/SV: TCF3/PAX5
FCGBP.D4873N
PIK3CD.H526>QA
WNT10B.R115*
WHSC1.E1099K
RYR2.R2805H

Supplementary Figure 23. Clonal architecture of diagnostic (D) and relapsed (R) tumors for PARIAD.

A) Scatter plot of mutant allele fraction (MAF) of diploid SNVs. B) At diagnosis, *TCF3/PAX5* structural variations along with a mutation cluster labeled by *FCGBP* p.Asp8473Asn were present in the ancestral founder clone. The founder clone has two descendant subclones, *WNT10B* p.Arg115Ter and *PIK3CD* p.His526>GlnAla. Only subclone *WNT10B* p.Arg115Ter survived the therapy and acquired *WHSC* p.Glu1099Lys in relapse, which subsequently gave rise to subclone *RYR2* p.Arg2805His.

SUPPLEMENTARY REFERENCES

1. Li, H. & Durbin, R. Fast and accurate short read alignment with Burrows-Wheeler transform. *Bioinformatics* **25**, 1754-60 (2009).
2. Li, H. *et al.* The Sequence Alignment/Map format and SAMtools. *Bioinformatics* **25**, 2078-9 (2009).
3. DePristo, M.A. *et al.* A framework for variation discovery and genotyping using next-generation DNA sequencing data. *Nat Genet* **43**, 491-8 (2011).
4. Zhang, J. *et al.* A novel retinoblastoma therapy from genomic and epigenetic analyses. *Nature* **481**, 329-34 (2012).
5. Zhang, J. *et al.* The genetic basis of early T-cell precursor acute lymphoblastic leukaemia. *Nature* **481**, 157-63 (2012).
6. Edmonson, M.N. *et al.* Bambino: a variant detector and alignment viewer for next-generation sequencing data in the SAM/BAM format. *Bioinformatics* **27**, 865-6 (2011).
7. Wang, J. *et al.* CREST maps somatic structural variation in cancer genomes with base-pair resolution. *Nat Methods* **8**, 652-4 (2011).
8. Mullighan, C.G. *et al.* Genomic analysis of the clonal origins of relapsed acute lymphoblastic leukemia. *Science* **322**, 1377-80 (2008).
9. Pounds, S. *et al.* Reference alignment of SNP microarray signals for copy number analysis of tumors. *Bioinformatics* **25**, 315-21 (2009).
10. Venkatraman, E.S. & Olshen, A.B. A faster circular binary segmentation algorithm for the analysis of array CGH data. *Bioinformatics* **23**, 657-63 (2007).
